# Optimisation of pipes with constant diameter using the heuristic optimality criterion

**DOI:** 10.12688/openreseurope.15943.2

**Published:** 2026-04-09

**Authors:** David Blacher, Michael Harasek

**Affiliations:** 1 LKR Light Metals Technologies, AIT Austrian Institute of Technology, Lamprechtshausener Straße 61, Ranshofen, 5282, Austria; 2 Institute of Chemical, Environmental and Bioscience Engineering, TU Wien, Getreidemarkt 9/166, Vienna, 1060, Austria

**Keywords:** Lattice Boltzmann method, shape optimization, heuristic optimality criterion, laminar internal fluid flow, pressure drop minimization, Cellular Automaton

## Abstract

**Background:**

Minimising internal pressure drop in pipes is crucial for energy efficiency of fluid flow applications. Numerous computational optimisation tools that are capable of modifying flow geometries to reduce the pressure drop have been developed. Among these is a comparably simple heuristic optimisation al- gorithm which mimics erosion and sedimentation processes based on the shear stress in the vicinity of the domain boundaries. Although this method succeeds in modifying flow geometries for reduced pressure drop, it allows the fluid domain to widen during the reshaping process. Therefore, a reported reduction of pressure drop is not only caused by an improvement of the flow path, but also by an increase in the domain width. However, pipes with a constant circular diameter are favoured in many applications because they can be easily manufactured.

**Methods:**

Here we combine the heuristic optimisation approach with a novel geometrical constraint that maintains constant average diameter throughout the reshaping process. To our knowledge, this is the first application of a diameter-preserving constraint to the heuristic optimality criterion, enabling assessment of pressure drop reduction achieved solely through flow path modification without diameter dilation effects. We determined the applicability of the new algorithm for 2D channel and 3D pipe ge- ometries, conducting numerical simulations using the Lattice Boltzmann method with Reynolds numbers ranging from 40 to 500.

**Results:**

For the novel constant diameter constraint, the method successfully derived improved shapes across most tested Reynolds numbers while maintaining the initial average diameter. Notably, shapes derived at *Re* = 40 unexpectedly outperformed those derived at higher Reynolds numbers across all tested flow conditions, suggesting that low-Re geometries may capture fundamental flow features beneficial across wider Reynolds number ranges.

**Conclusions:**

This finding suggests applicability to higher Reynolds number flows, potentially even at turbulent industrial flows to be investigated in fu- ture research.

## Plain language summary

A high pressure drop in pipes limits the energy efficiency of machines, and there are computer tools that can change the pipe design to reduce the pressure drop. One method mimics erosion and sedimentation and is called heuristic optimality criterion. However, this method widens the pipe during the design process. This is problematic, because pipes with varying widths are harder to be produced. In this article, we describe how to restrict the reshaping method to leave the pipe width unchanged. We tested this method using computer simulations for 2D channels and 3D pipes with flows of different behaviours described by their Reynolds number. The Reynolds number describes the flow behaviour—whether it is smooth (laminar) or chaotic (turbulent). We successfully improved pipe shapes while keeping the diameter fixed across all tested conditions. Interestingly, we found that shapes designed for the lowest Reynolds number (Re=40) performed better than shapes specifically designed for higher Reynolds numbers. This unexpected result suggests that simple shapes derived at low flow speeds might work well across a wide range of conditions, potentially even for turbulent flows in industrial applications—an exciting direction for future research.

**Table 1.** Mathematical symbols frequently used. Bold letters represent vectors (e.g. ***r***). Capital letters with two subscript indices represent second-order tensors (e.g. *S*_*ij*_), lower-case letters their elements (e.g. *S*_*ij*_). Symbols with a tilde (e.g. $\tilde{t}$ ) denote dimensionless quantities.

Symbol	Unit	Meaning
*∆p*	1	Relative pressure drop
δ *t*	1	Numerical time step
ρ	kg m ^−3^	Volumetric mass density
$\xi_{i}$	m s ^−1^	Particle velocity set of lattice model
*d*	m	Diameter
*d*_0_	m	Initial diameter
*f _e_*	1	Fraction of eroding nodes
*f _s_*	1	Fraction of sedimenting nodes
*t _a_*	t	Adaptation time
*f _i_* ( ***r***, ***ξ***, *t*)	s ^3^ m ^−6^	Particle distribution function
*M*	1	Surface area in voxel units
*p*	N m ^−2^	Pressure
***r***	m	Position
*Re*	1	Reynolds number
*S*_*ij*_	s ^−1^	Strain rate tensor
|| *S*_*i,j*_||	s ^−2^	Frobenius norm of the strain rate tensor
*u*_0_	m s ^−1^	Reference velocity
*V*	m ^3^	Fluid volume

## 1. Introduction

### 1.1 State of the art

Fluid flow applications such as heat exchangers, fluid distributors and gas ducts must be carefully designed to minimise pressure drop in order to maximise energy efficiency. Many approaches to optimise geometries exist, such as the adjoint ^1^^,^^2^ or conjugate gradient ^3^ method. These methods are capable of finding solutions for mathematical optimisation problems and allow for the combination of multiple objective- and cost-functions, making these approaches appropriate for a wide range of applications, including for turbulent flows ^4^ and the design of flow manifolds. ^5^ However, a geometry optimised in such a way might not always be feasible or economically viable because of practical, geometrical or material-specific restrictions. Adding such constraints in a convenient mathematical form can be challenging and requires an experienced user with holistic knowledge about fluid dynamics, mathematical optimisation and the unique demands of the application. Wang et al. ^6^ proposed a different reshaping algorithm based on a relatively simple “heuristic optimality criterion”. The shape of an initial flow geometry is modified in an iterative process: first, the fluid flow through the geometry is simulated using the Lattice Boltzmann Method (LBM). Then, the shape is slightly altered, mimicking erosion and sedimentation depending on the current flow field. These steps are repeated until a minimum pressure drop hence as optimum is found. The reshaping is governed by the following rules, implemented as a cellular automaton (CA):

• The fluid volume is kept constant throughout the reshaping process.
• The geometry only changes at the fluid-solid interface.
• Erosion occurs where viscous stress near wall nodes is highest. The neighbouring solid nodes become fluid.
• Sedimentation is applied to fluid nodes with the smallest value of dynamic pressure.

Wang et al. tested their method on 2D elbows and bifurcations and found a considerable reduction of pressure drop compared to the initial shapes across a range of Reynolds numbers from *Re* = 0.0267 to *Re* = 80. The applicability of the method was then proven by Wang et al. for the design of a fluid distributor. ^7^ Later, Tarlet et al. made use of the same method to derive a suitable geometry for a fluid mixer. ^8^ A similar approach was taken by Park et al., ^9^ but they chose the strain rate as criterion for both erosion and sedimentation. Park et al. tested their method on 2D and 3D rectangular ducts at Reynolds numbers up to *Re* = 100. The resulting 2D elbow shapes are in close accordance with those Wang et al. published. Tao et al. ^10^ also implemented the heuristic optimality criterion but they combined it with additional optimisation approaches. They start with an empty box as initial fluid domain, within which an optimal flow can form unhindered by wall boundaries. Then, the fluid domain is shrunken by substituting fluid cells with solid zones where dynamic pressure is lowest until a certain volume is reached. During a second phase, the shape is further enhanced by erosion and sedimentation, with the volume being held constant similar to the heuristic optimality criterion as proposed by Wang et al. Unfortunately, Tao et al. did not specify how this volume was chosen in their publication, which is why a direct comparison of the results is non-trivial. Interestingly, Tao et al. adapted the method for ANSYS Fluent, a finite volume based solver. While the use of a finite volume based solver has some advantages over the Lattice Boltzmann Method (LBM see section subsection 2.1) such as fast convergence for steady-state simulations, it requires meshing of the fluid domain after each reshaping iteration. This time-consuming operation is not necessary when coupling a reshaping algorithm with the LBM.

### 1.2 Motivation - constant diameter constraint

The incompressible Navier-Stokes equations are simulated for a Newtonian fluid under isothermal conditions with isotropic, homogeneous and constant physical properties. This idealization is representative of many practical fluids such as water and air in moderate temperature ranges. Pipe elbow geometries are ubiquitous in fluid flow applications including heat exchangers, fluid distributors, and gas ducts in industrial systems, where pressure drop minimization directly impacts energy efficiency. The heuristic optimality criterion method was originally developed for laminar flows. This publication validates our implementation against the existing publications by Wang et al. and Park et al. before investigating the novel constant diameter constraint across Reynolds numbers from 40 to 500, which span the laminar flow regime.

While many industrial fluid flow applications—including air ducts, fluid distributors, and heat exchangers—operate in the turbulent regime at significantly higher Reynolds numbers, geometries derived for laminar flows may still provide performance benefits. Wang et al. successfully applied shapes derived at low Reynolds numbers to practical industrial applications operating at higher flow rates. Our findings show that shapes derived at *Re* = 40 unexpectedly outperform those derived at *Re* = 100–500 for their respective flow conditions ( Section 4.4), suggesting that low-Re shape derivation may capture fundamental geometric features beneficial across a wider Reynolds number range—potentially extending into the turbulent regime. Investigating whether the constant diameter constraint enhances this transferability represents an interesting direction for future work.

The methods developed by Wang et al., ^6^ Park et al. ^9^ and Tao et al. ^10^ offer simple ways to improve flow geometries. However, there is a major drawback limiting the applicability of the resulting geometries: in the presented cases, applying the reshaping algorithm results in a widening of the fluid domain diameter. This is because the path between inlet and outlet becomes shorter as the reshaping is applied. Due to the constant fluid volume constraint, the accumulating surplus of fluid nodes causes a widened cross section. This also implies that the reduction of pressure drop reported in the course of the reshaping was not solely caused by an improvement of the flow path, but also by the increased domain width. Further increasing the width would likely cause the pressure drop to reduce even more. However, these geometries would not be favoured in real life applications where the diameter of pipes is often limited by a number of factors: available space, requirements regarding the stability of the pipes and material costs limit the possible size of the geometry. Moreover, the manufacturing process of pipes with varying diameter would require advanced techniques. Therefore, a new geometrical constraint is presented to counteract the diameter dilation. This novel constraint represents the key contribution of this work: unlike previous implementations by Wang et al., Park et al., and Tao et al., which allowed diameter variation, our method actively maintains constant average diameter. This decouples the pressure drop reduction due to flow path optimization from the trivial reduction achievable through diameter increase, enabling proper assessment when pipe diameter is constrained by space, structural requirements, or manufacturing considerations. We validate our implementation through numerical agreement with Wang et al. and Park et al. for a standard test case before investigating the constant diameter constraint. In the following, the Lattice Boltzmann Method (LBM) is briefly introduced. Then, the numerical setup is presented followed by a detailed description of the reshaping algorithm and the new reshaping constraint. The optimisation method was implemented as cellular automaton (CA) into the existing open-source LBM library called Palabos. ^11^

## 2. Mathematical model

### 2.1 Lattice Boltzmann Method

**Figure 1.**
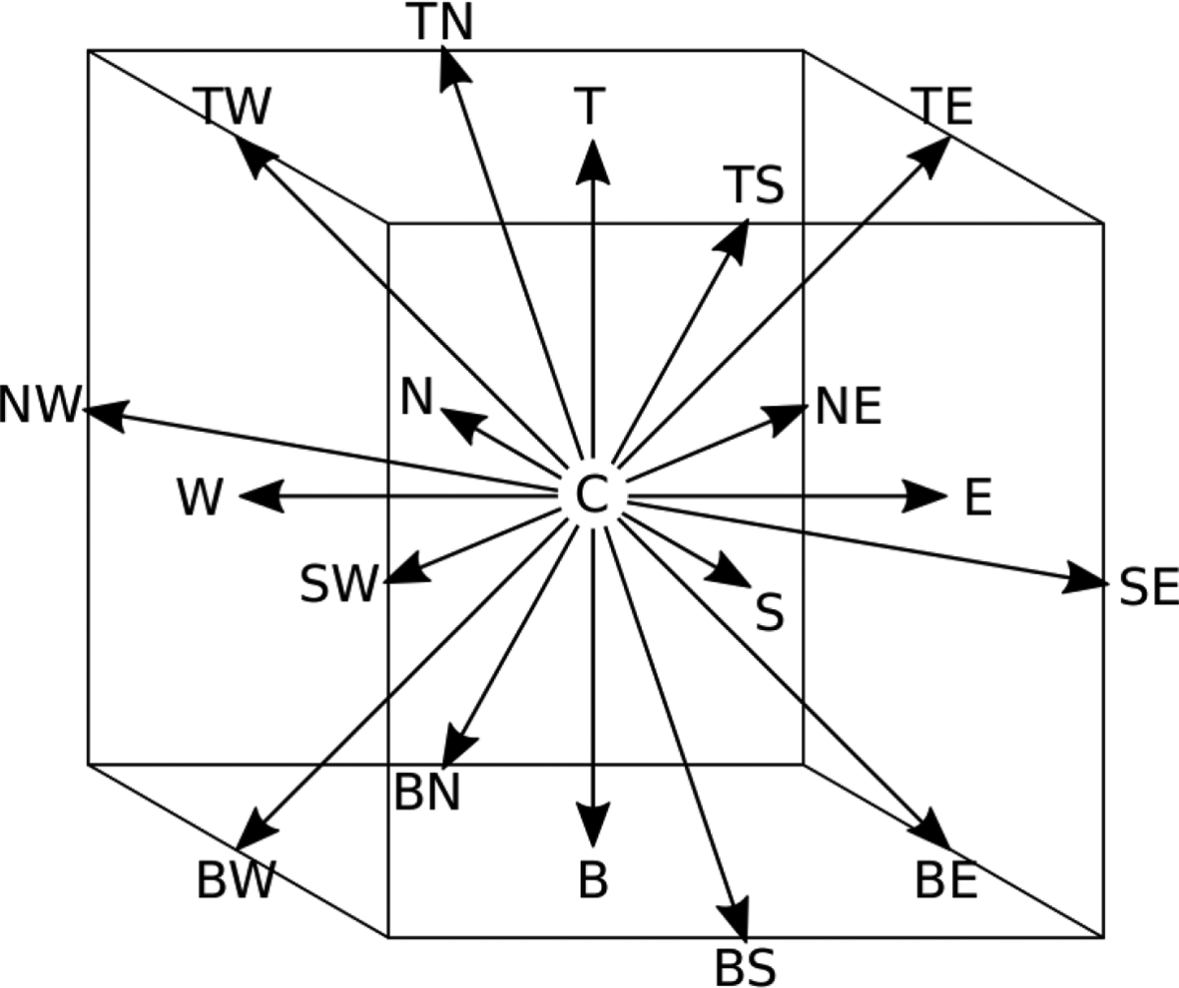
Discrete velocities of the D3Q19 lattice. To help visualise the phase space, each velocity is referred to by letters indicating its direction: “N,E,S,W,T,B,C” stand for “North, East, South, West, Top, Bottom, Center”.

An elaborate description of the lattice Boltzmann method (LBM) can be found for example in ^12^ on which this short introduction is based on. The LBM is a numerical model used for fluid flow simulations. It models the incompressible Navier-Stokes equations by solving a discretised Boltzmann transport equation, describing the evolution of the particle distribution function *f _i_* due to particle interactions. The particle population *f _i_* at each node ***r*** describes the number of particles moving with one velocity ***ξ**_i_* out of a set of possible velocities. This set is designed in such a way that particles move from one node to a neighbouring node exactly within one numerical time step. For reasons of numerical stability and accuracy, a 3-dimensional model with 19 velocities (D3Q19) was used for all our test cases. The corresponding set of velocities ***ξ _i_*** and their weights *w _i_* of the D3Q19 lattice model are described by Figure 1 and Table 2. Aside from particles at rest ( *i* = 0), all other velocities ( *i* ∈ [1, 18]) point towards one of 18 neighbouring nodes. During one time step δ *t*, the particles at each node *f _i_* ( ***r***) collide and then stream to neighbouring nodes:

$$f_{i}\left(r+\xi_{i}\mathrm{\delta t},t+\mathrm{\delta t}\right)=f_{i}\left(r,t\right)+\Omega_{i}\left(r,t\right)$$ (1)

**Table 2.** Velocities ***ξ**_i_* and weights *w _i_* for the D3Q19 model. Velocities are scaled by the lattice velocity *u _lb_.* The notation ( *x*, *y*, *z*) *_c_* indicates all possible permutations of the given tuples.

*ξ _i_*	*w _i_*	*i* ∈
(0, 0, 0)	1 */*3	{ C }
(± *u _lb_*, 0, 0) *_c_*	1 */*18	{ E, N, W, S, T, B }
(± *u _lb_*, ± *u _lb_*, 0) *_c_*	1 */*36	{ NE, NW, SE, SW, TE, TW, BE, BW, TN, TS, BN, BS }

For practical reasons, Equation 1 is separated in two computational steps. First the post-collision populations *f _i_^c^* are calculated at the current location ***r***:

$$f_{i}^{c}\left(r,t+\mathrm{\delta t}\right)=f_{i}\left(r,t\right)-\frac{1}{\tau }\left(f_{i}-f_{i}^{\mathrm{eq}}\right)$$ (2)

Then, the populations at ech node are updated based on the incoming particles from neighbouring nodes:

$$f_{i}\left(r+\xi_{i}\mathrm{\delta t},t+\mathrm{\delta t}\right)=f_{i}^{c}\left(r,t+\mathrm{\delta t}\right)$$ (3)

In Equation 2, the post-collision particle interaction *f _i_^c^* is calculated by approximating the collision operator *Ω* as local relaxation towards equilibrium, the lattice Maxwell-Boltzmann distribution $f_{i}^{\mathrm{eq}}$. This is known as the BGK model.

$$f_{i}^{\mathrm{eq}}\left(r\right)=\rho w_{i}\left[1+\frac{1}{c_{s}^{2}}\left(\xi_{i}\cdot u\right)+\frac{1}{2c_{s}^{4}}{\left(\xi_{i}\cdot u\right)}^{2}-\frac{1}{2c_{s}^{2}}\left(u\cdot u\right)\right]$$ (4)

The relaxation time τ depends on the chosen lattice viscosity $\nu_{\mathrm{lb}}$.

$$c_{s}=\frac{1}{\sqrt{3}}\;\tau =\frac{\nu_{\mathrm{lb}}}{c_{s}^{2}}+1/2$$ (5)

From the distribution function *f _i_*, all relevant macroscopic flow properties can be calculated:

$$\rho =\sum_{i=0}f_{i}\;p=c_{s}^{2}\cdot \rho$$ (6)

$$u=\frac{1}{\rho }\sum_{i=0}^{q}f_{i}\xi_{i}\;S_{\mathrm{ij}}=-\frac{1}{2\tau c_{s}^{2}\rho }\sum_{i=0}\left(f_{i}-f_{i}^{\left(\mathrm{eq}\right)}\right)\xi_{i}\xi_{i}$$ (7)

The incompressible fluid is simulated under isothermal conditions, with isotropic, homogeneous and constant physical properties. The simulations conducted for this paper utilised regularised BGK dynamics, a multiple relaxation time (MRT) model. Latt et al. ^13^ showed that this enhanced model can reproduce analytically known solutions more accurately than the basic BGK model by restoring symmetry properties of the populations before every collision step. The LBM is particularly well-suited for shape optimization applications due to its natural handling of complex, evolving geometries without remeshing and straightforward implementation of boundary conditions on irregular surfaces. Table 3 lists the numerical parameters of the simulations for each chosen Reynolds number ( *Re*). To ensure comparability with results by Wang et al. and Park et al., the resolution was set to the same value, given by the number of nodes across the characteristic length (the inlet diameter) *N*_*cells*,0_ = 1/δ _*x*_ = 40. This resolution choice prioritizes direct methodological comparison—enabling isolation of the constant diameter constraint effects—rather than grid convergence studies.

**Table 3.** Chosen numerical parameters given in lattice units. The maximum acceptable lattice viscosity $\nu_{\mathrm{lb}}^{\max}$ based on the Mach limit is provided for reference.

*Re*	Velocity *u _lb_*	Viscosity *ν _lb_*	*N*_*cells*,0_ = 1/δ *_x_*	$\nu_{\mathrm{lb}}^{\max}$
40	0.04	0.04	40	0.0577
100	0.05	0.02	40	0.0231
200	0.05	0.01	40	0.0115
400	0.05	0.005	40	0.0058
500	0.05	0.004	40	0.0046

### 2.2 Numerical parameter selection

The choice of numerical parameters for LBM simulations requires careful consideration of several constraints to ensure stability and accuracy while maintaining computational efficiency. For a given Reynolds number *Re* and spatial resolu- tion δ *_x_*, the lattice viscosity $\nu_{\mathrm{lb}}$ must be chosen to satisfy the Mach number constraint, which requires flow velocities to remain well below the lattice speed of sound ( *Ma* = *u _lb_*/ *c _s_* < 0.1) for the incompressible LBM formulation.

Simulating a flow with a specific Reynolds number at a given resolution δ _*x*_ leaves the lattice viscosity $\nu_{\mathrm{lb}}$ as the only flexible parameter to ensure the Mach limit is not violated. The Mach limit imposes an upper bound on the acceptable lattice viscosity. Starting from the Mach number constraint and the definition of lattice viscosity, we derive:

$$\mathrm{Ma}=\frac{u_{\mathrm{lb}}}{c_{s}}=\frac{\delta_{t}}{\delta_{x}c_{s}}\leq {\mathrm{Ma}}^{\max}$$ (8)

$$\to \delta_{t}\leq {\mathrm{Ma}}^{\max}\delta_{x}c_{s}$$ (9)

Using the definition of lattice viscosity:

νlb=δtδx2Re (10)

→δt=νlbδx2Re (11)

Combining these expressions yields the maximum acceptable lattice viscosity:

⇒νlb≤Mamax⋅csδx⋅Re=0.13⋅δx⋅Re (12)

where *c _s_* = 1/ $\sqrt{3}$ is the lattice speed of sound for the D3Q19 model and *Ma ^max^* = 0.1. This relationship shows that higher Reynolds numbers require proportionally lower lattice viscosities to maintain numerical stability.

The relaxation time τ is related to the lattice viscosity through $\tau =\nu_{\mathrm{lb}}/c_{s}^{2}+1/2$ and must satisfy τ > 0.5 to ensure positive viscosity. Values of τ close to but above 0.5 can lead to numerical instabilities, while values significantly above 1 increase relaxation times. For the Reynolds numbers investigated ( *Re* = 40 to 500), the chosen parameters ( Table 3) maintain τ in a stable range while respecting the Mach limit.

The resolution of *N*_*cells*,0_ = 40 nodes across the inlet diameter was chosen to match Wang et al. ^6^ and Park et al., ^9^ enabling direct methodological comparison. More detailed considerations regarding the overall research design, including convergence criteria and adaptation time selection, are provided in the accompanying thesis. ^14^

### 2.3 Simulation setup

**Figure 2.**
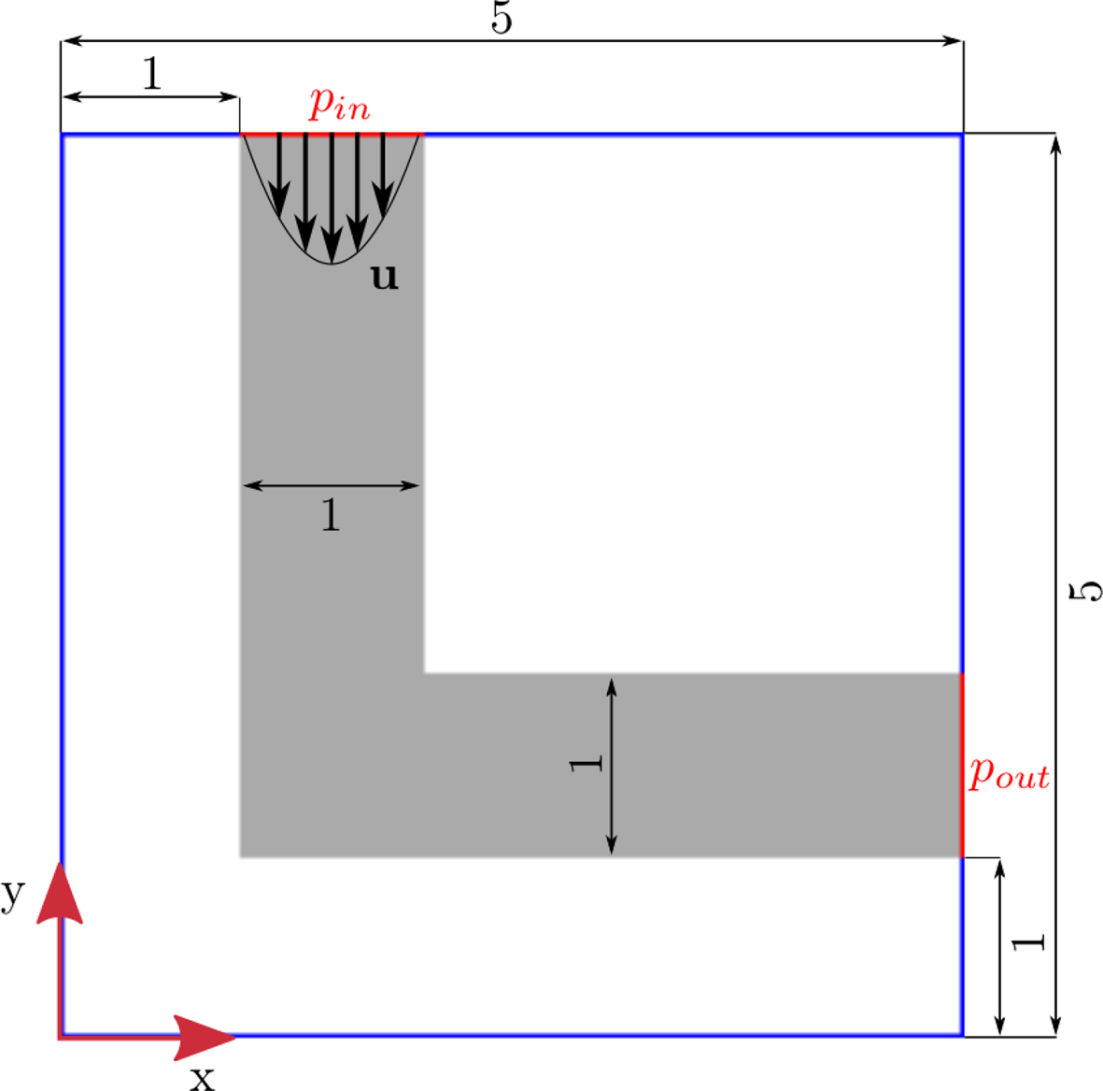
Initial 2D elbow geometry. Boundary conditions: prescribed velocity *u* at inlet; constant pressure at outlet. Red lines indicate where pressure is calculated for evaluation of pressure drop.

The initial pipe elbow geometry is created by two cylinders of the same diameter aligned perpendicularly to each other. One lies along the x-, the other along the y-axis. They extend from the inlet and outlet to the diagonal plane where they intersect. The simulation domain is a cuboid comprised of *N _total_* = *N _x_ · N _y_ · N _z_* nodes. See Table 4 for the size of the domain for each geometry. The XY-plane at *z* = *N _z_*/2 of the initial 3D elbow corresponds to the initial 2D elbow shape (see Figure 2). In the 2D simulations, a channel with infinite height is simulated by applying periodic boundary conditions in z-direction. At the outlet, a constant pressure condition ${\tilde{p}}_{\text{out}}=1$ is imposed. This serves as a reference pressure (zero gauge pressure), allowing the flow to exit freely while enabling calculation of the pressure drop across the elbow geometry.

**Table 4.** Optimisation parameters. Adaptation time between reshaping iterations ${\tilde{t}}_{a}$ is given as dimensionless value. The fraction of wall-neighbouring nodes subject to erosion in each iteration is denoted as *f _e_.* The domain size consists of the total number of computational cells Nx,Ny,Nz.

	Optimisation		Domain size		
Type	${\tilde{t}}_{a}$	*f _e_*	Nx	Ny	Nz
2D channel	15	0.02	200	200	4
3D pipe	25	0.01	400	320	120

At the inlet, a parabolic velocity profile is imposed, representing a fully developed laminar flow condition characteristic of Poiseuille flow. This ensures the flow entering the elbow is representative of practical pipe flow conditions where flow has developed in an upstream section. The no-slip condition at solid walls is enforced through bounce-back dynamics, physically representing the zero velocity at the fluid-solid interface observed in viscous flows. Before the reshaping algorithm is applied, the flow field is initialised: starting from zero, the inlet velocity increases step-wise until it reaches the reference velocity ${\tilde{u}}_{0}=1$ in its centre. The flow is further simulated until the flow field converges to a steady state. Convergence is determined by monitoring the global change of kinetic energy—when this change falls below a specific threshold, the flow is considered to have reached steady state and reshaping may begin. This criterion ensures the initial flow field is fully developed before optimization starts. Between each reshaping iteration, the fluid flow is also simulated for an adaptation time ${\tilde{t}}_{a}$ . This is necessary for the flow field to adapt to the modified geometry. Ideally, each reshaping step would be executed only after reaching a new steady state. However, this approach would require an impractical amount of computing time. Instead, a sufficient adaptation period was determined by analyzing the convergence of the wall shear stress distribution after a single reshaping step. This led to the choice of approximately 1.5 times the time needed for one flush through the geometry—long enough for the flow field to adapt adequately, yet computationally efficient. The chosen adaptation times for each geometry are listed in Table 4. This approach is more efficient than re-initializing the flow from zero velocity after each reshaping step, as done by Wang et al. ^6^ and Park et al., ^9^ because the shape changes only incrementally between iterations.

## 3. Method

The individual steps of the optimisation procedure will be addressed in the following. As a reference, refer to Figure 5 which describes the implemented algorithm in a flowchart.

### 3.1 Erosion and sedimentation model

The reshaping algorithm allows modifications of the fluid domain only at the fluid-solid interface. Fluid nodes along the interface will be referred to as wall-neighbouring nodes in the remaining text. The reshaping is guided by a CA based on the strain rate. To determine which of the wall-neighbouring nodes should be affected by the reshaping, they are ranked by the local value of || *S*_*i*, *j*_||, the Frobenius norm of the strain rate tensor. Sedimentation will be applied to a fraction *f _s_* of wall-neighbouring nodes where || *S*_*i*, *j*_|| is smallest. Erosion will be applied to a fraction *f _e_* of solid nodes directly neighbouring the fluid nodes which suffer from the greatest stress || *S*_*i*, *j*_||. An eroding solid node joins the fluid domain by changing its dynamics from bounce-back to BGK. The resulting new fluid node is initialised with zero velocity, which is physically appropriate because these nodes are direct neighbors of solid walls subject to the no-slip condition. During the subsequent adaptation time ${\tilde{t}}_{a}$ , the flow field adjusts to the new geometry and the initial zero velocity is replaced within a few time steps as the flow adapts. This simple initialization is both physically reasonable and computationally stable. If a fluid node sediments, it becomes a bounce-back node. In order to change the speed of the entire optimisation process, the fractions of affected nodes can be scaled: allowing more nodes to erode and to sediment in each step reduces the number of overall reshaping steps required to reach an optimised shape. However, Wang et al. recommend to choose a small fraction of affected nodes in order to ensure the shape actually converges to its optimum. Wang et al. chose the fraction of nodes affected by either erosion or sedimentation ( *f _e_* + *f _s_*) as 0.04 of all wall-neighbouring interface nodes *n _f_.* Park et al. varied the fraction between 0.02 and 0.05 in their simulations. The fractions of eroding nodes chosen for the present study is listed in Table 4. The ratio of eroding nodes to sedimenting nodes *f _e_*/ *f _s_* determines if the fluid geometry may grow or shrink in each iteration. This balance is governed by the chosen reshaping constraint: either keeping the overall fluid volume or the geometry diameter constant throughout the reshaping. In order to keep the fluid volume constant, the total number of fluid nodes must not change, requiring *f _e_*/ *f _s_* = 1 throughout the entire optimisation process. A constant geometry diameter can be achieved by adjusting the ratio of eroding nodes to sedimenting nodes during the reshaping process.

### 3.2 Calculation of current diameter

The fluid volume *V* is calculated as the sum of all fluid nodes:

$$V=n_{\text{fluid}}\cdot V_{n}=n_{\text{fluid}}$$ (13)

where *n _fluid_* is the number of fluid nodes and *V _n_* = 1 is the volume per node in lattice units.

**Figure 3.**
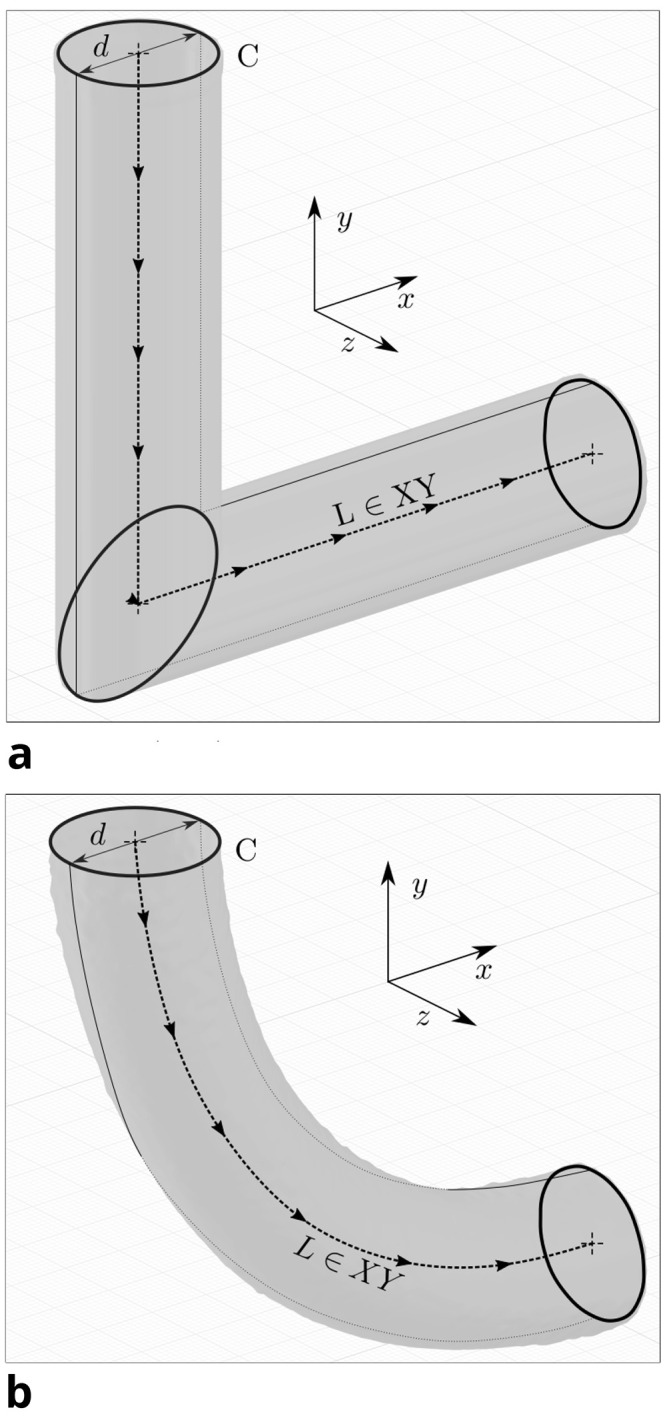
Geometrical analysis: by tracing figure F (enclosed by curve C) along the path L (which lies in the XY-plane), the 3D pipe geometry can be generated. (a) Initial geometry. Curve C is depicted 3 times: at the inlet, the intersection of cylinders and at the outlet. (b) Optimised geometry for *Re* = 40. Curve C is depicted 2 times: at the inlet and outlet.

At each iteration, the diameter is evaluated by considering the relationship between volume and surface area of the fluid domain. This is based on a generalisation of the theorem of Pappus, ^15^ which allows the calculation of surface area and volume for a solid generated by moving a plane figure F (enclosed by curve C) perpendicularly along an arbitrary curve L. Figure 3 depicts how both the initial and an exemplary optimised pipe geometry can be described as such a solid. Its volume *V* equals the product of the area *A* of figure F and distance *l _F_* travelled by the centroid of F:

$$V=A\cdot l_{F}$$ (14)

Provided the traced curve L lies in a plane, the generated body’s surface area *M* equals the product of the arc length *s* of C and the distance *l _C_* travelled by the geometric centroid of C:

$$M=s\cdot l_{C}$$ (15)

Consequently, we can write the ratio of a body’s volume to its surface area as:

$$V/M=\frac{A\cdot l_{F}}{s\cdot l_{C}}$$ (16)

For the simple cases of figure F being either a circle or rectangle, the centroids of figure F and curve C coincide, and *l _F_* = *l _C_* = *l*, resulting in:

$$\Rightarrow V/M=\frac{A}{s}$$ (17)

For a circle with radius *r* = *d*/2 we get:

$$V/M_{○}=\frac{r^{2}\pi }{2\mathrm{\pi r}}=\frac{r}{2}=\frac{d}{4}$$ (18)

This corresponds to a pipe with circular cross section. A channel with infinite height (quasi 2D case) can be approximated as a slender ( *h* >> *d*) rectangle with width *d* and height *h*:

$$V/M_{○}=\frac{\mathrm{dh}}{2\left(d+h\right)}=\frac{d}{2\left(\frac{d}{h}+1\right)}\approx \frac{d}{2}$$ (19)

In both cases, the ratio *V*/ *M* is proportional to the diameter *d*, but independent of the curve. Therefore, the average diameter of these geometries can be gauged by *V*/ *M* even if the path *l* changes. This calculation method is not suited for complex geometries, such as bifurcations or 3D geometries with non-circular cross sections. For the conducted simulations, the change of the diameter relative to its initial value *d*/ *d*_0_ was of interest. This ratio can be calculated with knowledge of the initial values of volume *V*_0_ and surface area *M*_0_ and their current values *V* and *M*:

$$\frac{V/M}{V_{0}/M_{0}}=\frac{d}{d_{0}}=\frac{\tilde{d}}{{\tilde{d}}_{0}}$$ (20)

In dimensionless values the initial diameter ${\tilde{d}}_{0}$ is 1, and the current diameter is calculated as

$$\to \;\tilde{d}=\frac{V/M}{V_{0}/M_{0}}$$ (21)

The volume *V* is approximated by the sum of all fluid nodes, with a volume of *V _n_* = 1 each: The surface area *M* is calculated from a generated smoothed STL representation of the fluid-solid interface. ^16^ The STL file format is commonly used to store a 3D representation of a surface comprised of unstructured triangulated sub-surfaces, each defined by its unit normal and vertices.

### 3.3 Adjusting the erosion/sedimentation ratio for a constant diameter

The measurement of the diameter allows to adjust the erosion/sedimentation ratio to counteract shrinking or widening during the reshaping process. Each reshaping step consists of the erosion process, a re-evaluation of the diameter and, if necessary, one or more layers of sedimentation. Sedimentation is applied incrementally until the diameter reaches its initial value. The number of sedimenting nodes for each increment is proportional to the current deviation of the diameter from its initial value:

$$f_{s}={\left(\frac{d}{d_{0}}\right)}^{3}-1\;\text{for}\;3D\;\text{pipe}$$ (22)

$$f_{s}={\left(\frac{d}{d_{0}}\right)}^{2}-1\;\text{for}\;2D\;\text{channel}$$ (23)

**Figure 4.**
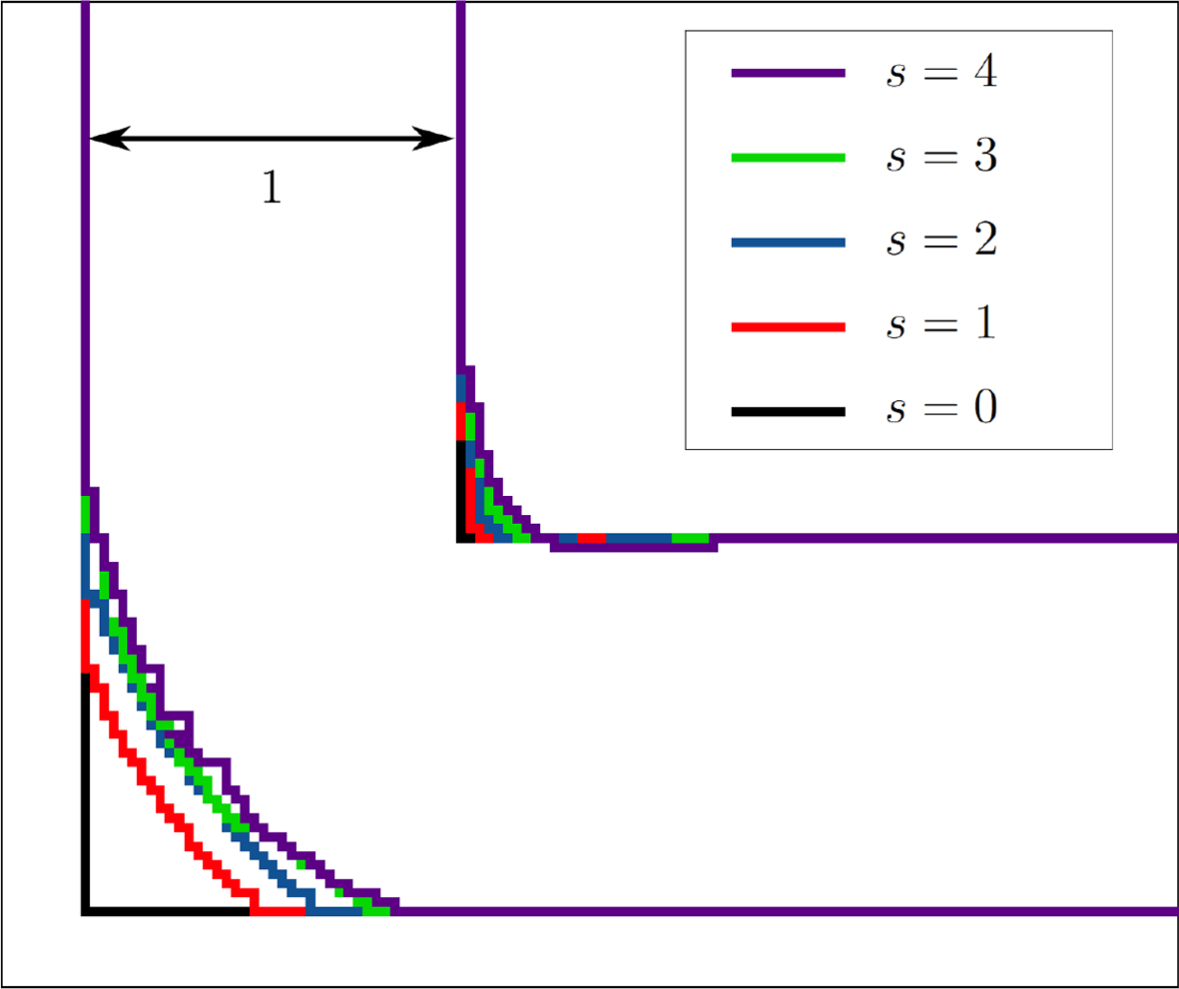
Reshaping of the 2D elbow channel. During the first two reshaping steps, multiple layers sediment. In the subsequent steps, only few multi-layer sedimentation steps take place.

This ensures convergence of the diameter to its initial value. In order to allow regions with little fluid flow to sediment first, a new layer of solid nodes can be added on top of the last with each increment. Because the fluid-solid interface changes each time, a new ranking by local strain rate || *S*_*i*, *j*_|| is required to determine the nodes for the next increment. However, as no fluid flow is simulated between these incremental sedimentation steps, the ranking of the new wall- neighbouring nodes is based on strain rate information of the previous reshaping step. Figure 4 shows how the initial hard edge of the elbow geometry becomes rounded during the first reshaping step. Such a sedimentation of multiple layers occurs mainly in the first few reshaping steps.

**Figure 5.**
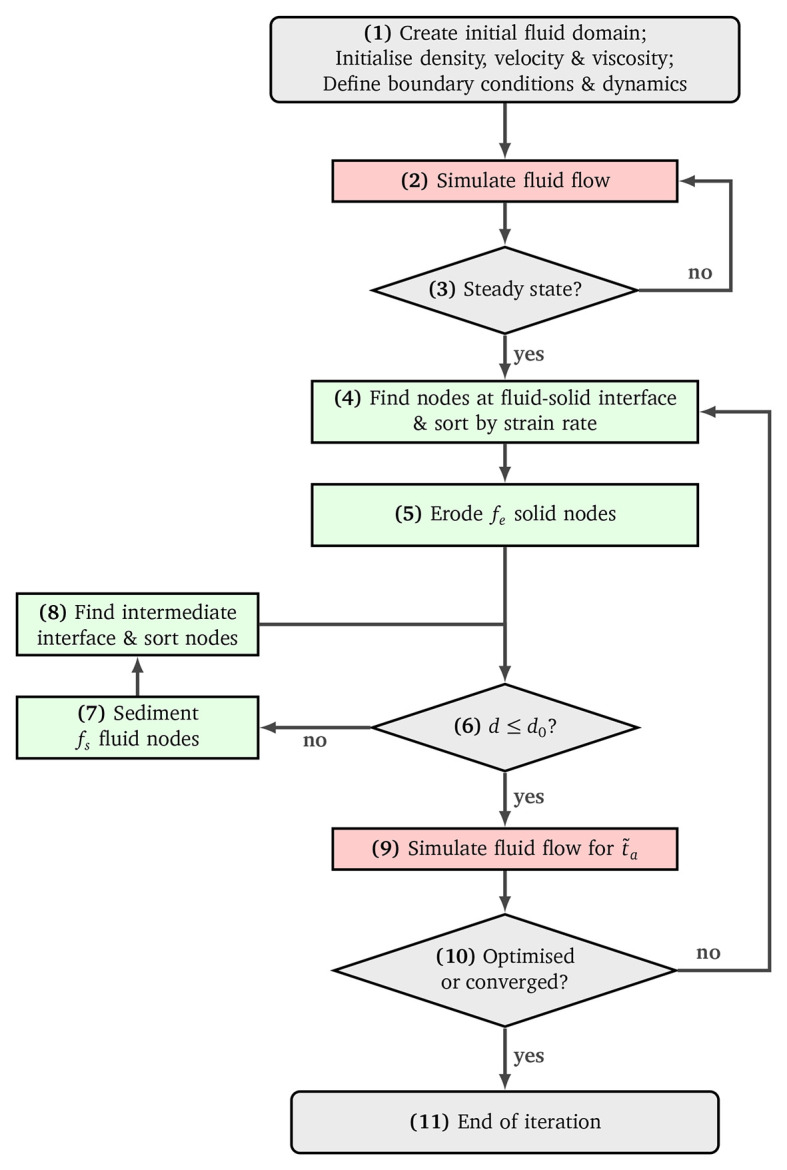
Flowchart of the reshaping algorithm.

### 3.4 Pressure drop evaluation

The inlet pressure ${\tilde{p}}_{\text{in}}$ was calculated as the average at the inlet domain boundary. The outlet pressure is defined as the reference pressure ${\tilde{p}}_{\text{out}}=1$ . The dimensionless absolute pressure drop $\Delta {\tilde{p}}_{a}$ of a given fluid flow is the difference between inlet and outlet:

$$\Delta {\tilde{p}}_{a}={\tilde{p}}_{\text{in}}-{\tilde{p}}_{\text{out}}={\tilde{p}}_{\text{in}}-1$$ (24)

To allow the comparison of the pressure drop reduction in simulations with different Reynolds numbers, the relative pressure drop was calculated. The relative pressure drop for a given shape is the ratio of its current absolute pressure drop and the absolute pressure drop of the initial elbow shape.

$$\Delta p=\frac{\Delta {\tilde{p}}_{a}}{\Delta {\tilde{p}}_{a,\text{init}}}$$ (25)

By this definition, the optimisation of any shape started at a relative pressure drop of *∆p* = 1. If the pressure drop continuously increased after hitting a minimum or the shape converged to a final shape, the simulation was terminated and the shape yielding the best result exported.

## 4. Results and discussion

### 4.1 Validation against literature

To validate the implementation of the heuristic optimality criterion, the 2D channel elbow was optimized at *Re* = 40 under the constant volume constraint using identical numerical parameters as Wang et al. ^6^ and Park et al. ^9^

**Figure 6.**
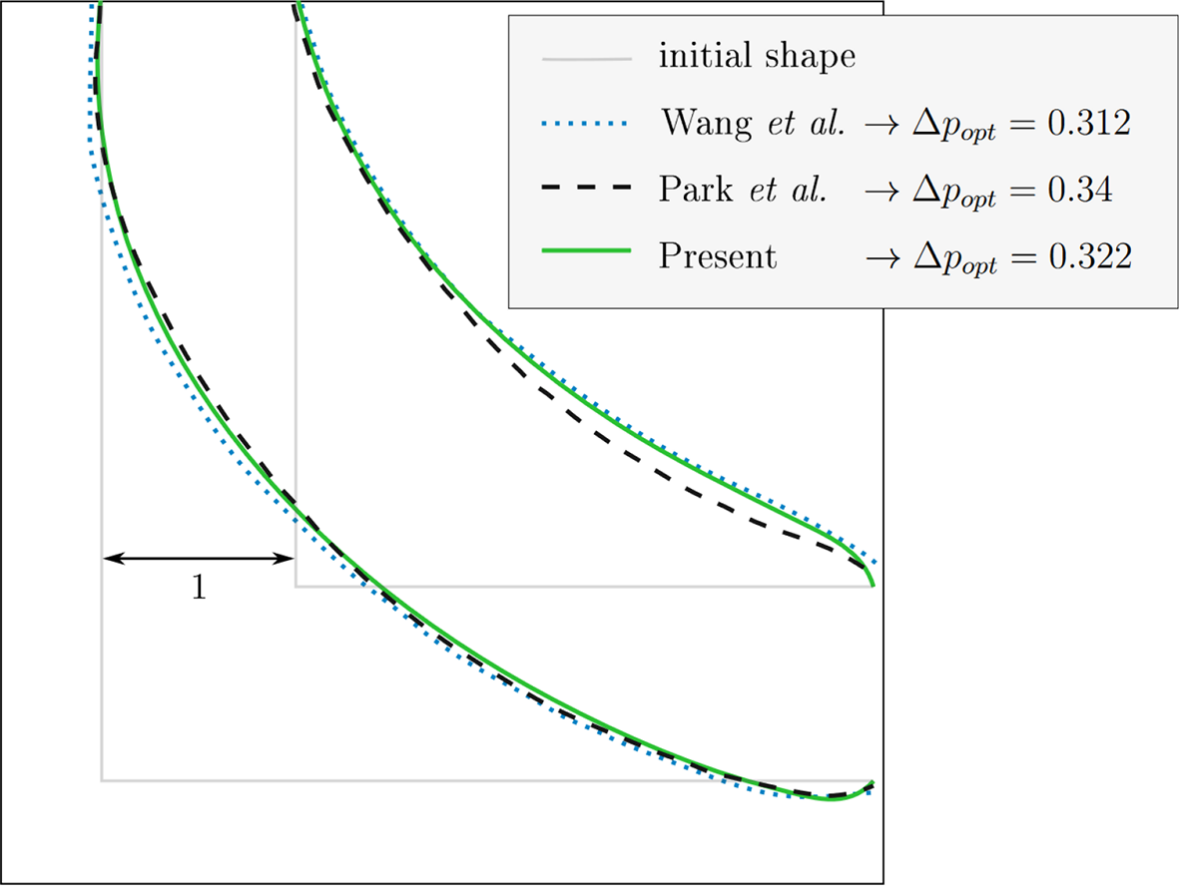
2D elbow: optimised shape at *Re* = 40 for constant volume. Comparison with existing publications. ^6^ ^,^ ^9^ Our result is depicted here and in Figure 7a as solid green line.

Figure 6 compares the optimized shapes. Our method achieved a pressure drop reduction to *∆p _opt_* = 0.322 (67.8% of the initial value), in excellent agreement with Wang et al. (68.8%) and Park et al. (66%). This close numerical agreement across three independent implementations confirms the correct application of the heuristic optimality criterion and validates the LBM simulation approach. This established baseline enables confident investigation of the novel constant diameter constraint introduced in this work.

The agreement also demonstrates that our choice of adaptation time and velocity initialization approach produces results consistent with the original methods, despite using a different flow field update strategy between reshaping steps.

Table 5 summarises the pressure drop achieved during the reshaping process. For all tested setups, shapes derived under the constant volume constraint yielded a smaller pressure drop at a wider diameter.

**Table 5.** Results of conducted simulations: Setup parameters are listed on the left hand side. On the right hand side, information on the optimised shape are given. Optimised pressure drop *∆p _opt_* and diameter *d* are relative to values at initial elbow shape. The number of the reshaping steps after which a minimum pressure drop was reached is listed as *s _opt_.* A ‘+’ symbol indicates that the shape was fully converged at this step.

Geometry	Constraint	*Re*	*∆p _opt_*	*d*	*s _opt_*	
2D channel	V	40	0.322	1.287	960	+
	V	200	0.130	1.240	605	+
	V	500	0.036	1.230	619	+
	d	40	0.739	1.001	379	
	d	200	0.472	1.002	771	+
	d	500	0.255	1.002	652	+
3D pipe	d	40	0.902	1.000	658	+
	d	100	0.972	1.000	125	
	d	200	0.943	1.000	38	
	d	400	0.832	1.000	147	

### 4.2 2D channel elbow

As validated in Section 4.1, our implementation produces results in excellent agreement with Wang et al. and Park et al. for the constant volume constraint. We now investigate the novel constant diameter constraint.

**Figure 7.**
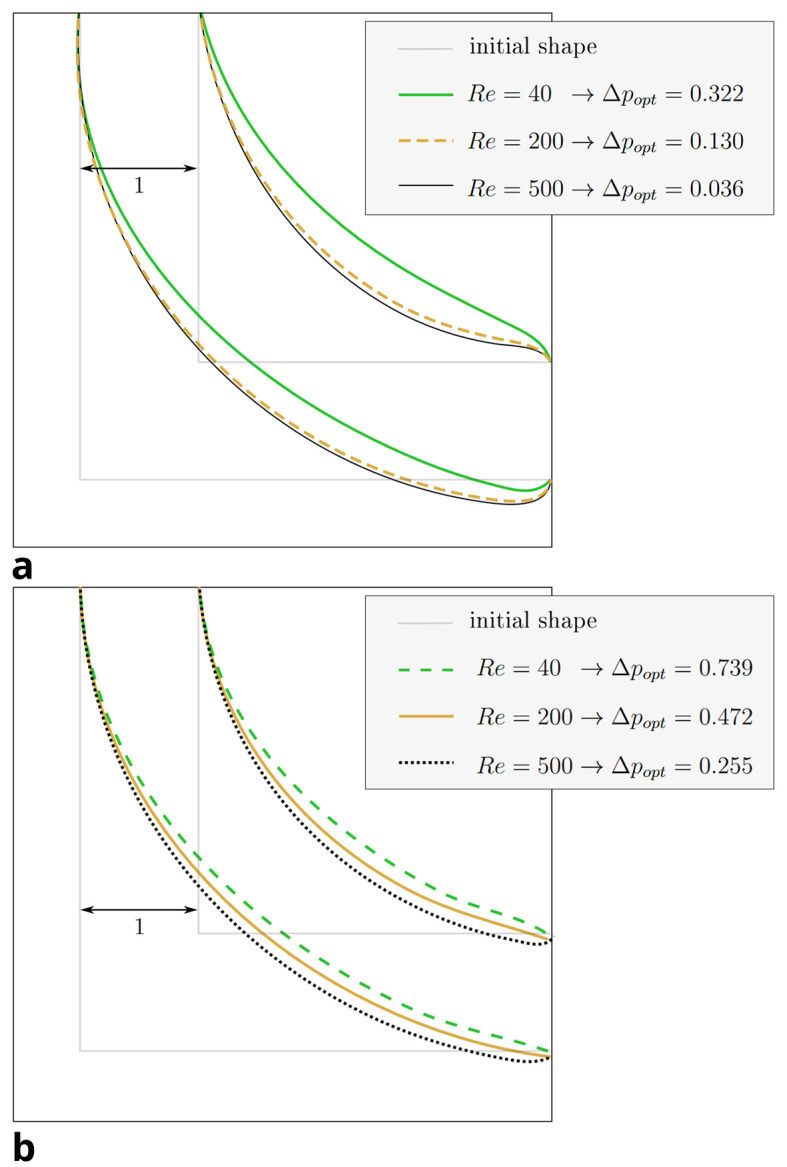
2D elbow: optimised shapes. (a) Shapes derived under constant volume constraint. (b) Shapes derived under constant diameter constraint.

**Figure 8.**
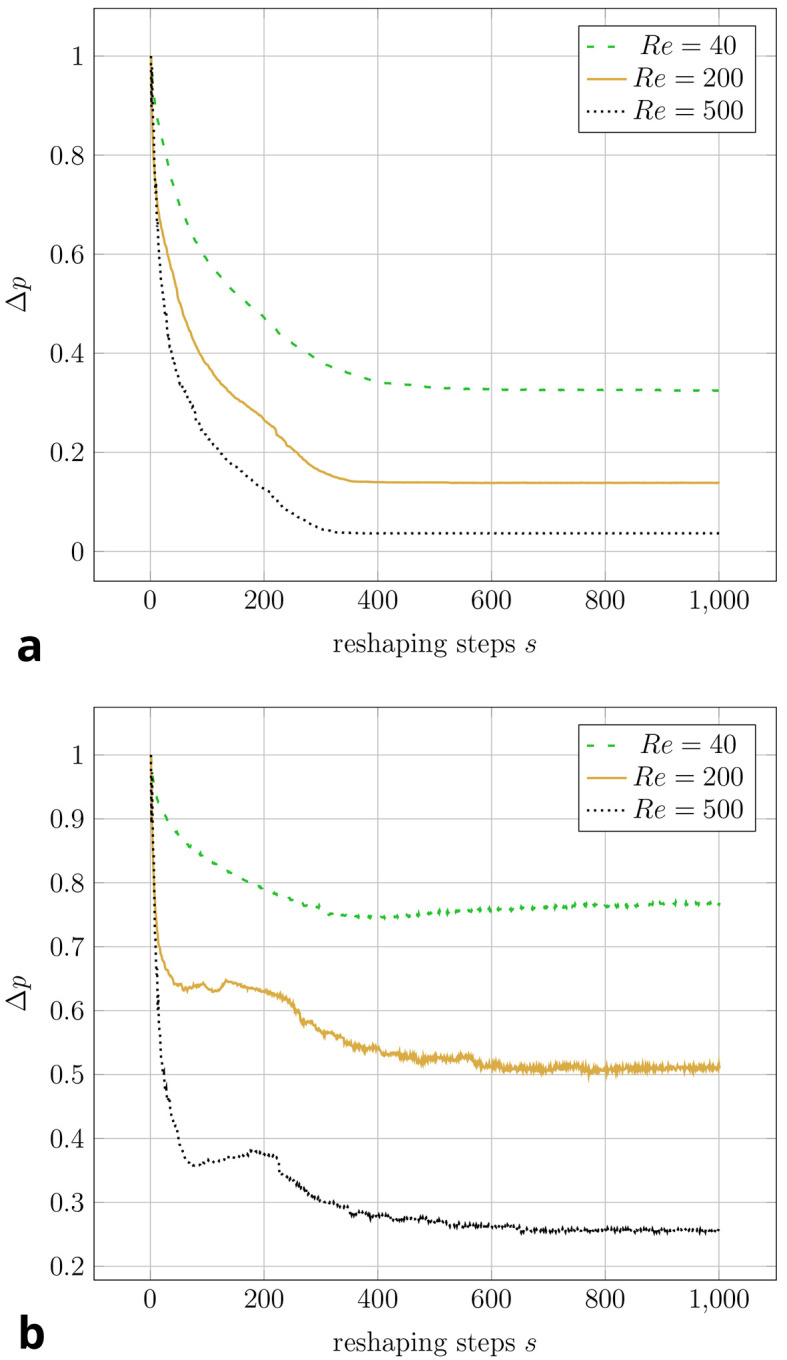
2D elbow: Evolution of relative pressure during the reshaping (at varying Reynolds numbers at either of the two reshaping constraints). For each simulation, the pressure drop values are relative to their initial values. Simulations at the same Reynolds number start with the same absolute pressure drop (the difference between inlet and outlet). (a) Constant volume constraint. (b) Constant diameter constraint.

**Figure 9.**
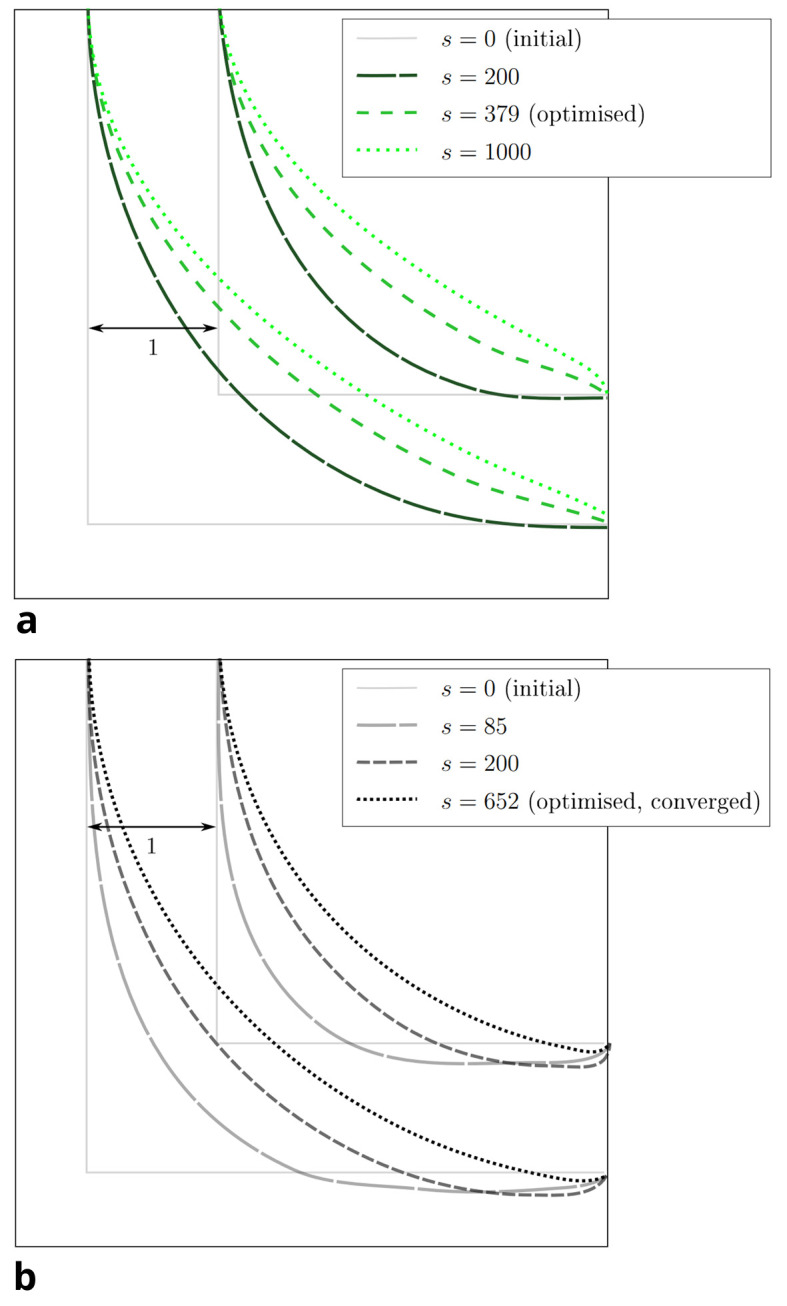
2D elbow: snapshots taken at selected reshaping steps *s* during reshaping. (a) Evolution of shape at *Re* = 40 and constant diameter. (b) Evolution of shape at *Re* = 500 and constant diameter.

Figure 8 visualises the change in pressure drop over the course of the reshaping for both constraints. In simulations with higher Reynolds numbers, an even smaller of *∆p _opt_* could be achieved. Figure 7 shows that the curvature of the resulting shapes increases with rising Reynolds number. This behaviour was also reported by Wang et al. across a range of lower Reynolds numbers. They found a straight connection between inlet and outlet for *Re* = 0.0267, and arc like shapes at *Re* = 20, 40, 80. In all but one of the simulations, the pressure drop converges to a minimum. Only during the optimisation at *Re* = 40 under the constant diameter constraint, *∆p _opt_* increased again by 2.6% after reaching a minimum at *s* = 379 reshaping steps. The evolution of shape during this simulation is depicted in Figure 9a. In all other 2D simulations, the geometry had reached a final shape and *∆p _opt_* its final value after approximately *s* = 500 reshaping iterations. The evolution of shape during one such simulation is depicted in Figure 9b. At *s* = 1000 reshaping iterations, the simulations were terminated.

### 4.3 3D pipe elbow

**Figure 10.**
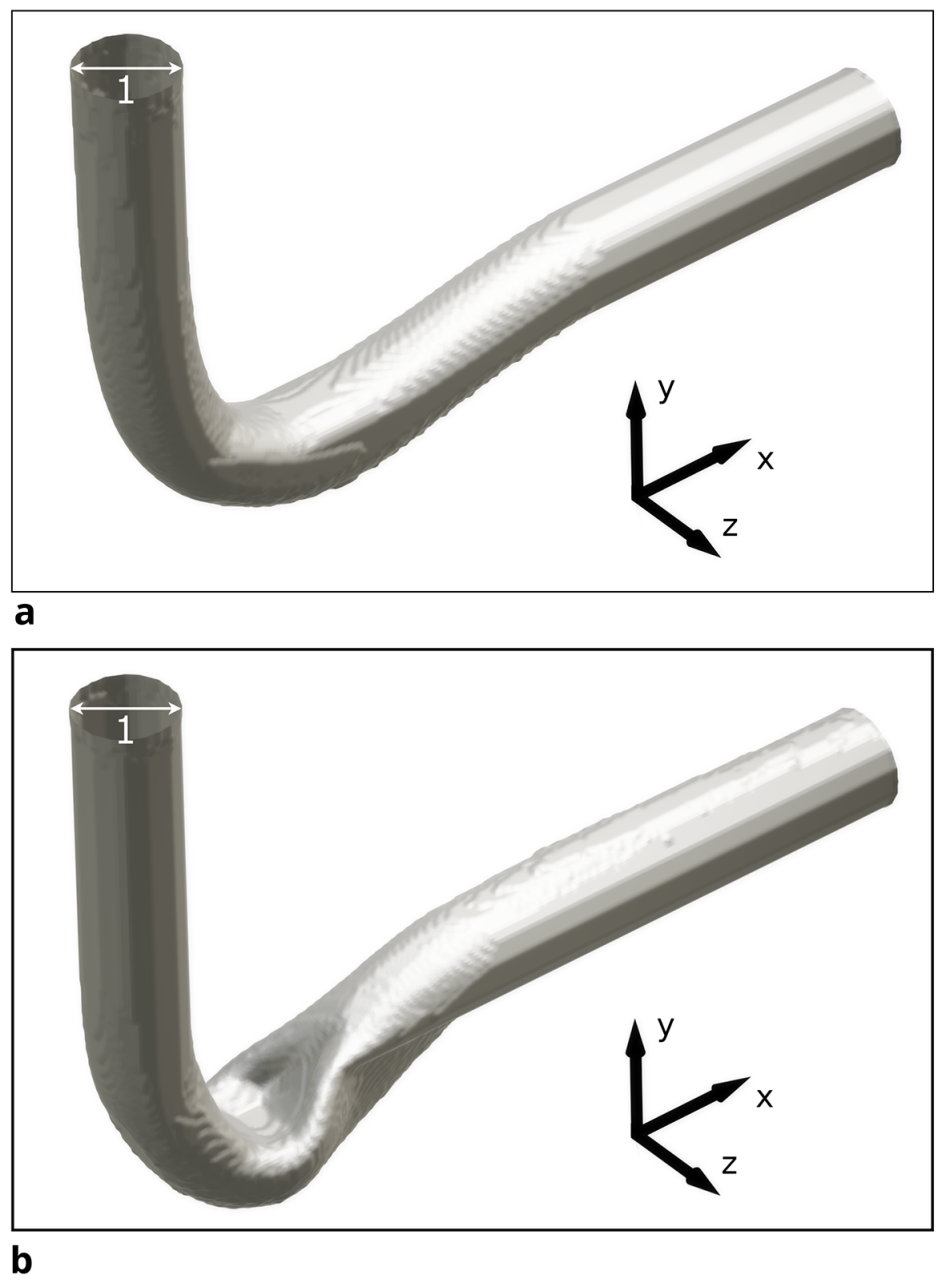
3D visualisation of pipe optimised at two Reynolds numbers. (a) *Re* = 100. (b) *Re* = 400. The pipe shows significant deformation.

**Figure 11.**
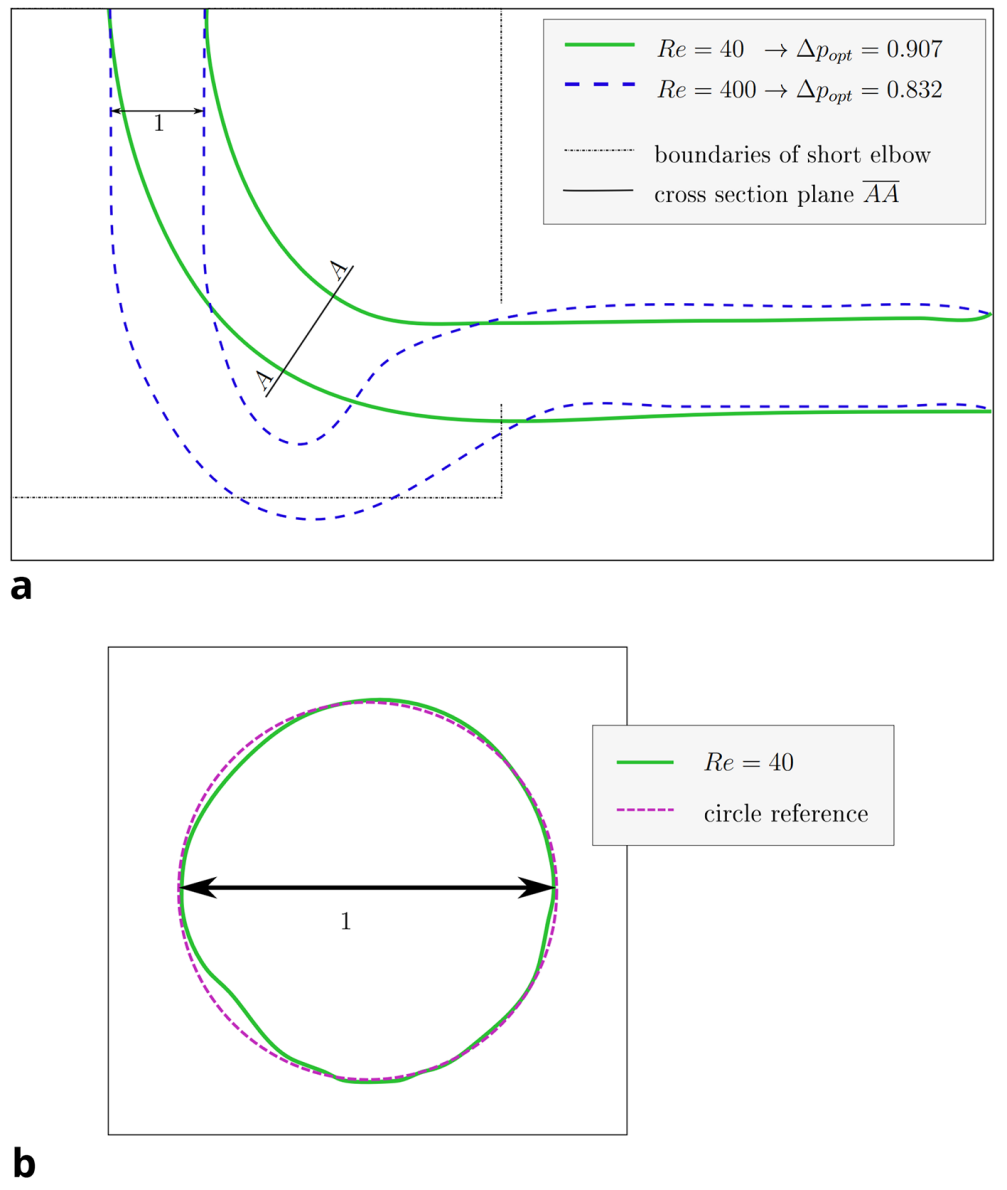
3D elbow: projections of shape derived at *Re* = 40. (a) XY-plane projection of shape derived at *Re* = 40. The cross section in plane $\bar{\mathrm{AA}}$ is depicted in figure b. The shape optimised at *Re* = 400 and the domain boundaries of the shorter 2D elbow are depicted for reference. (b) The cross section (plane $\bar{\mathrm{AA}}$ ) of the shape derived at *Re* = 40 retains an almost circular profile.

**Figure 12.**
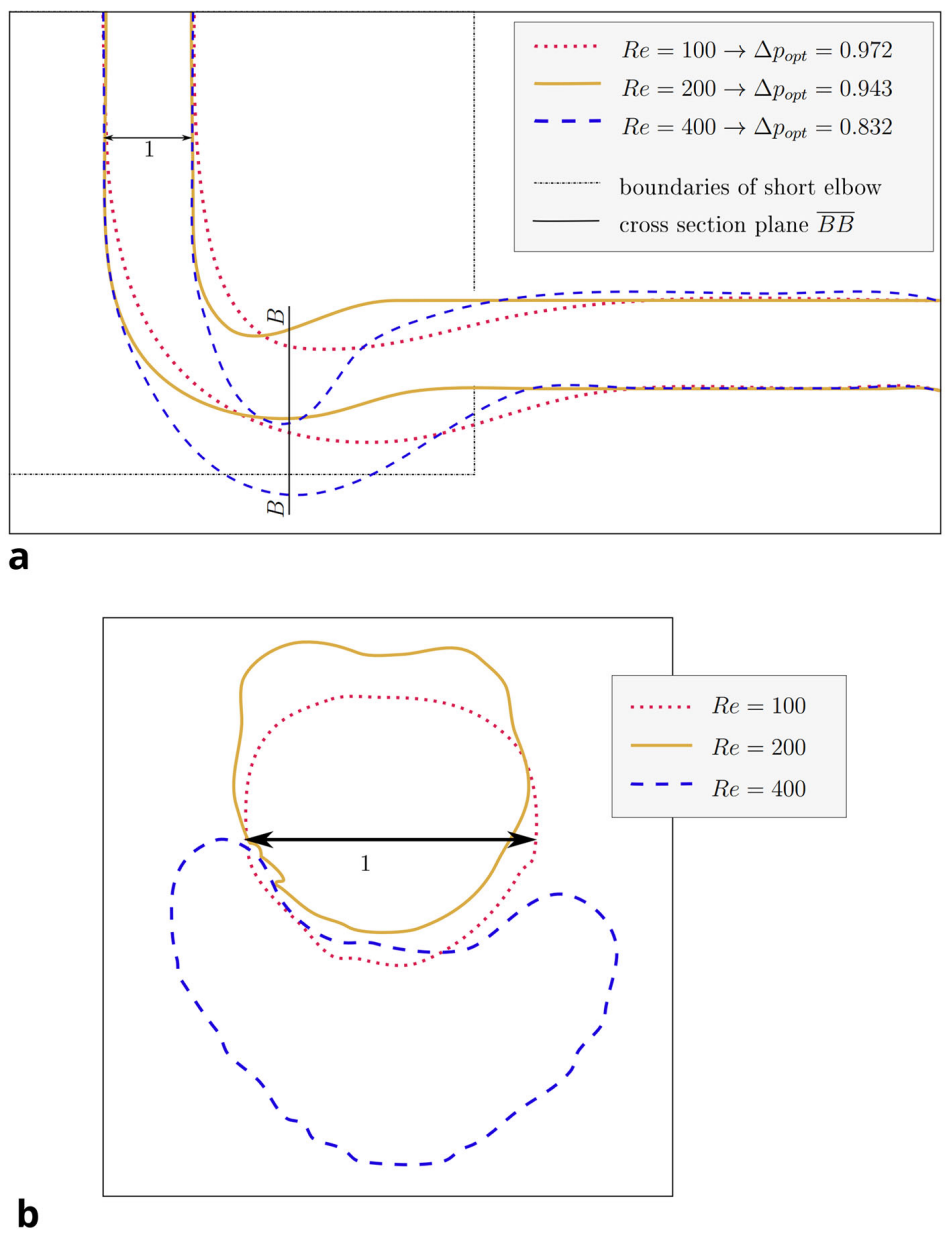
3D elbow: projections of shapes derived at *Re* ≥ 100. (a) XY-plane projection of shapes. The cross section in plane $\bar{\mathrm{BB}}$ is depicted in figure (b). The domain boundaries of the shorter 2D elbow are depicted for reference. (b) The cross sections of shapes in plane $\bar{\mathrm{BB}}$ (corresponds to YZ-plane at $\tilde{x}=3$ , equivalent to n _x_ = 120). With increasing Reynolds number, the deviation from a circular profile becomes more pronounced.

**Figure 13.**
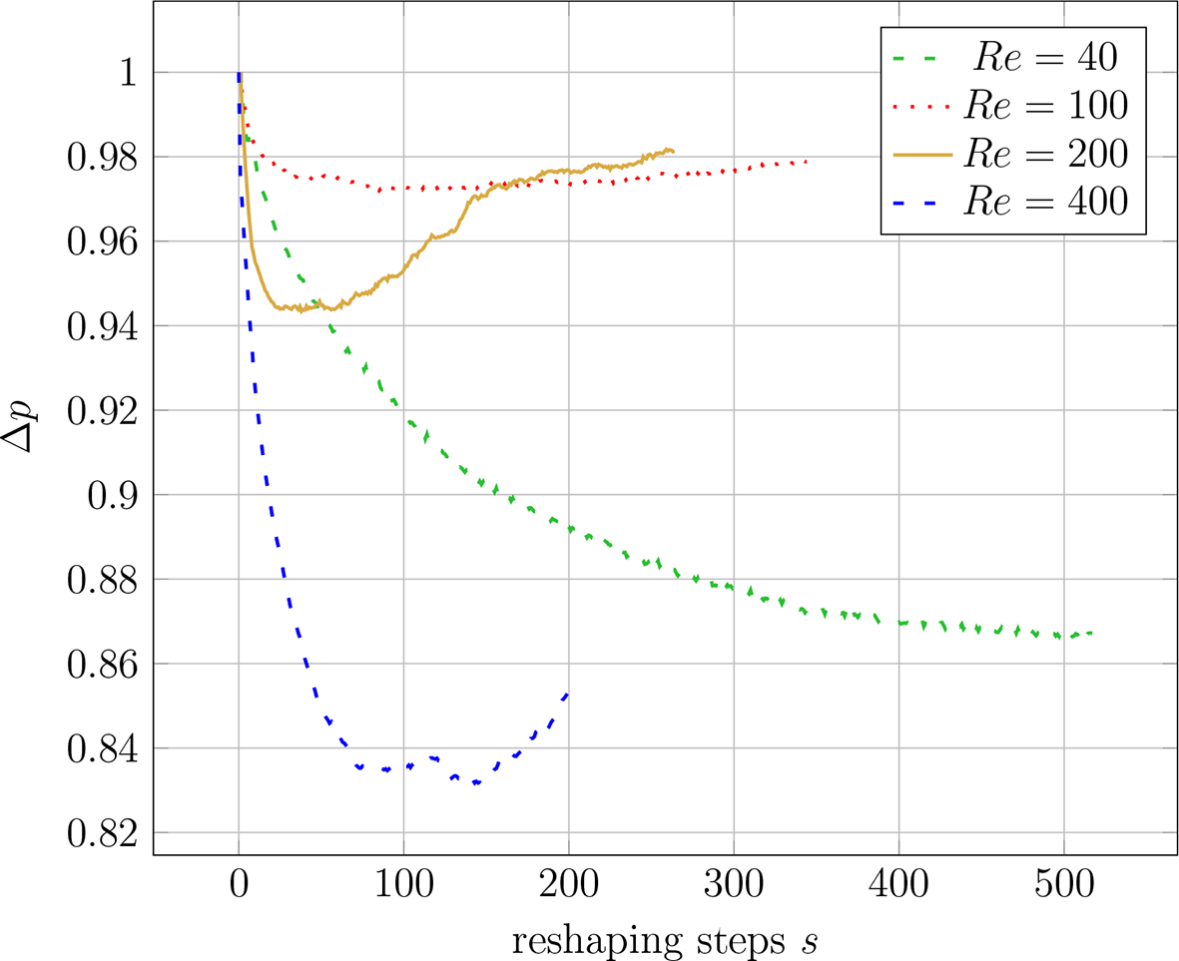
3D elbow: evolution of relative pressure drop during reshaping at different Reynolds numbers.

**Figure 14.**
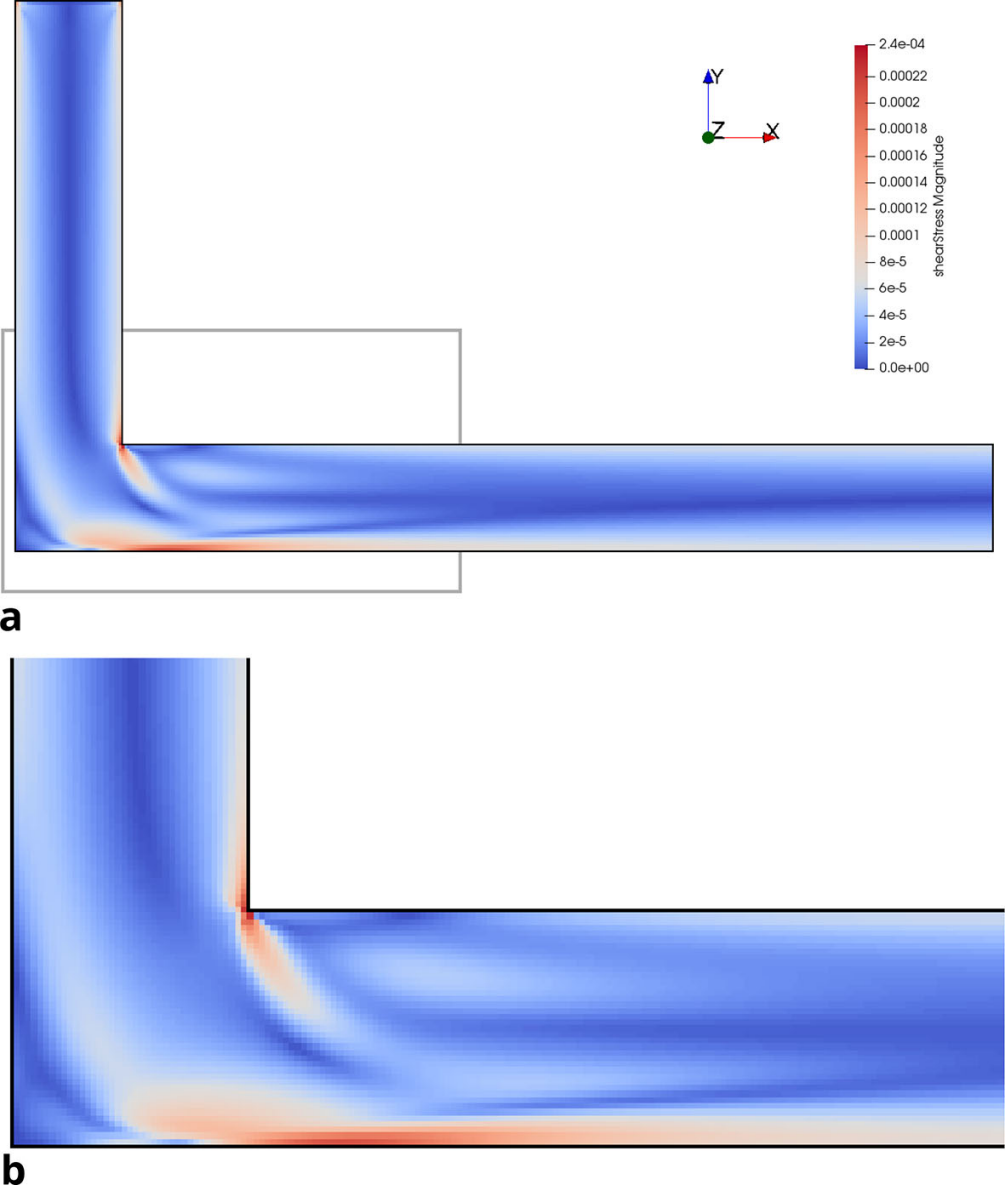
Shear stress in initial 3D pipe elbow at *Re* = 200. (a) Full geometry. Grey box indicates area of detailed view in figure below. (b) Detail of geometry.

**Figure 15.**
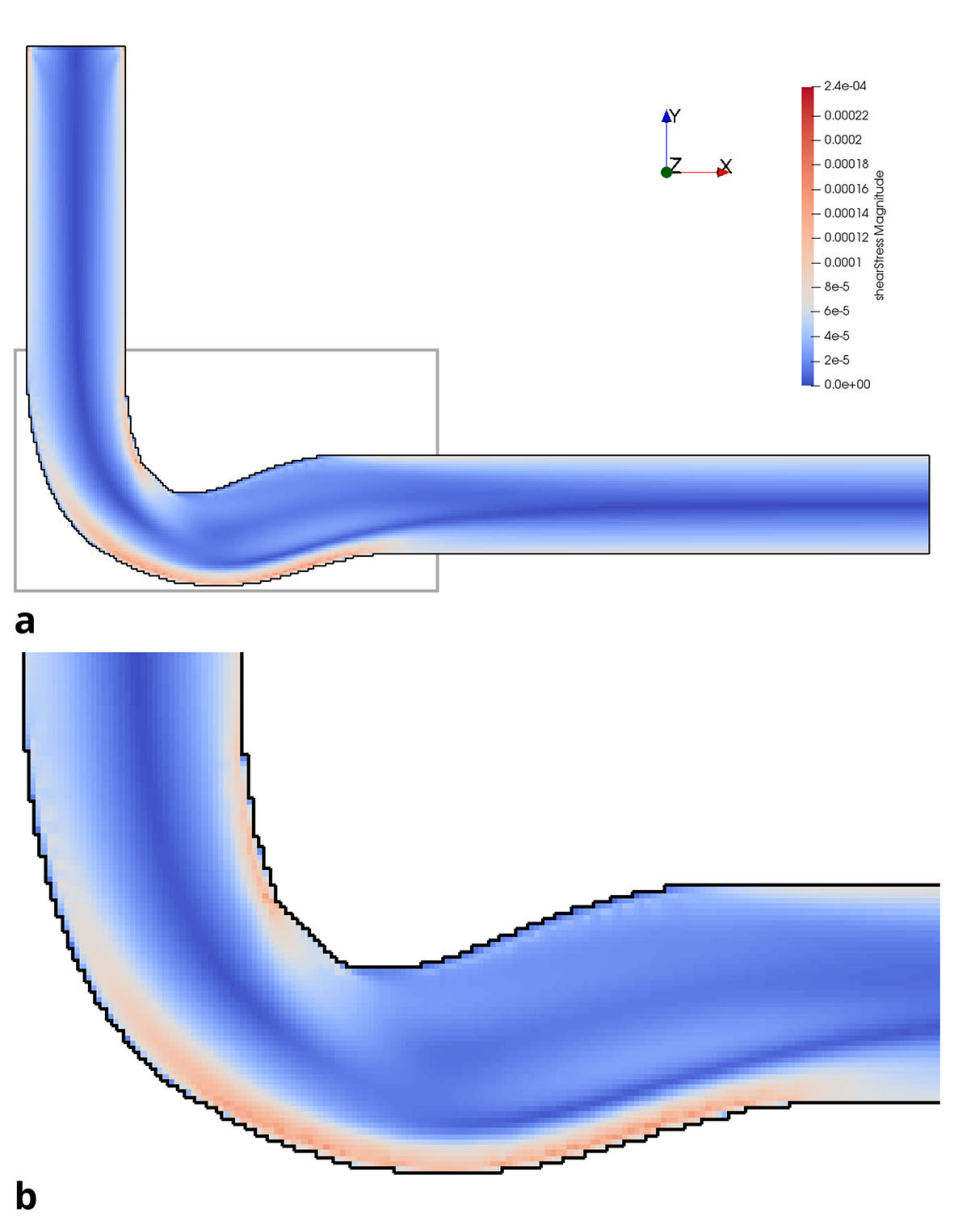
Shear stress in optimised 3D pipe elbow at *Re* = 200. (a) Full geometry. Grey box indicates area of detailed view in figure below. (b) Detail of geometry.

For the 3D pipe geometry, only reshaping with the constant diameter constraint was performed. Interestingly, the achieved relative pressure drop does not follow a clear trend. Figure 13 shows that *∆p* only converges to a minimum during the reshaping at *Re* = 40. To eliminate the influence of potential spatial limitations, a longer outlet was simulated by doubling the domain in the x-direction. 3D renderings of the derived shapes for *Re* = 100 and *Re* = 400 are visible in Figure 10. The shape derived at *Re* = 40 closely resembles those found in 2D at this Reynolds number (see Figure 11a): inlet and outlet are connected by an arc like shape. However, the shapes derived at *Re* 100 behave differently: the elbow dips below the level of the outlet, as visible in Figure 12a. Furthermore, the pipe cross section deviates from a circular profile: Figure 11b shows that for *Re* = 40, the cross section is still close to a circular profile. Above that, deviations increase with the Reynolds number, as visible in Figure 12b. The shear stress fields of the flow at *Re* = 200 in the initial and optimised 3D pipe are plotted in Figure 14 and Figure 15.

### 4.4 Convergence of shapes

For this study, the direct dependence of the shape optimisation on a variation of the initial geometry was not investigated, but some conclusions can still be drawn from the observed convergence behaviour. In any 3D simulation with *Re* ≥ 100, the shapes did not converge to reach a minimum pressure drop. Instead, an intermediate iteration with a minimum pressure drop could be identified by the algorithm. This is visible in Figure 13. Consequently, the intermediate shape with the lowest pressure drop could not have been returned if a subsequent iteration of the reshaping had been used as the initial shape. Therefore, there seems to be a dependency of the optimisation algorithm on the initial geometry for 3D geometries at *Re* ≥ 100 which demands to be explored in more detail. While Wang et al. ^6^ reported that the optimisation of 2D shapes at *Re* = 40 was largely unaffected by the initial geometry, this could not be verified for the reshaping with the new constant diameter constraint at *Re* = 40: here the smallest pressure drop was found after *s* = 379 iterations (see Table 5 and Figure 9a). For all other 2D cases, the geometry converged to a minimum. Overall, this indicates that the algorithm is indeed dependent on the starting geometry.

### 4.5 Quality of derived shapes

For both constraints and all setups (2D and 3D), shapes could be derived which yielded a reduced pressure drop com- pared to the initial geometries. The shapes derived at *Re* = 40 exhibit the shortest connection between in- and outlet. To rule out that these shapes perform better than the longer connections derived at *Re* ≥ 100, additional flow simulations were performed: for each tested Reynolds number *Re* ≥ 100, the flow through the reference shape derived at *Re* = 40 was analysed and *∆p*_*Re*=40_ calculated. The values are summarised in Table 6. Surprisingly, the reference shape derived at *Re* = 40 resulted in the lowest pressure drop for flows, across all tested Reynolds numbers. This behaviour was found both among the shapes derived with the constant diameter and the constant volume constraint. These findings indicate that the algorithm as adapted from Wang et al. and Park et al. encounters difficulty finding Reynolds-number-specific optima at *Re* ≥ 100. However, the superior performance of the *Re* = 40 shapes across all tested Reynolds numbers suggests an intriguing possibility: shapes derived using the heuristic optimality criterion at low Reynolds numbers may capture fundamental flow path features that remain beneficial at higher Reynolds numbers. If this transferability extends to turbulent flows—as Wang et al. demonstrated in practical applications—the heuristic optimality criterion combined with the constant diameter constraint could offer a computationally efficient approach to derive geometries applicable across a wide Reynolds number range, including turbulent industrial conditions. This hypothesis warrants investigation in future work.

**Table 6.** Comparing pressure drop *∆p* at a given Reynolds number through the dedicatedly derived shape and the shape derived at *Re* = 40.

	Flow at Re	Shape optimised at Re					*∆p _opt_*/ *∆p*_*Re*=40_
**2D channel**		40		200		500	
const. V	40	0.322		−−		−−	−−
	200	0.122		0.130		−−	1.065
	500	0.027		−−		0.036	1.335
const. d	40	0.739		−−		−−	−−
	200	0.452		0.472		−−	1.045
	500	0.206		−−		0.255	1.237
**3D pipe**		40	100	200	400		
const. d	40	0.902	−−	−−	−−		−−
	100	0.776	0.972	−−	−−		1.253
	200	0.746	−−	0.943	−−		1.265
	400	0.672	−−	−−	0.832		1.237

## 5. Conclusion

The aim of this research was to assess the potential pressure drop reduction by changing the fluid domain path without affecting its diameter at *Re* ≥ 40. This was partly achieved by combining the existing heuristic optimality criterion with a further constraint of keeping the average diameter constant. Simulations conducted using this procedure revealed some limitations of the algorithm: Firstly, the cross section of 3D pipes deviated from the initial circular profile at *Re* ≥ 100. Secondly, and more importantly, for any flow with *Re* ≥ 100, 2D and 3D shapes optimised specifically for this Reynolds number resulted in a higher pressure drop than a reference shape derived at *Re* = 40. This behaviour was observed not only for shapes derived with a fixed average diameter, but also when the fluid volume was kept constant, as proposed by Wang et al. ^6^ and Park et al. ^9^ We concluded that the capability of the heuristic optimality criterion to optimise the investigated initial shapes is limited at *Re* ≥ 100.

Future work should address several open questions. First, the physical mechanisms causing reduced performance at *Re* ≥ 100 merit investigation—is the wall shear stress criterion insufficient at higher Reynolds numbers where inertial effects dominate? Alternative criteria (pressure-based or multi-objective optimization) could be explored. More importantly, the unexpected finding that shapes derived at *Re* = 40 outperform Reynolds-number-specific designs at *Re* ≥ 100 opens an intriguing research direction: these geometries may also provide pressure drop reduction for turbulent flows at much higher Reynolds numbers. Many industrial applications—air ducts, fluid distributors, and heat exchangers—operate in the turbulent regime where direct simulation-based optimization becomes computationally prohibitive. If the transferability of low-Re derived geometries extends into the turbulent regime, the heuristic optimality criterion with the novel diameter constraint could offer a practical, computationally efficient design approach for industrial applications. Wang et al. successfully demonstrated such transfer in fluid distributor design; systematic investigation of this phenomenon, particularly with diameter-constrained optimization, represents a valuable avenue for future research with significant industrial relevance. Second, the method could be extended to other geometries (bifurcations, manifolds) and optimization objectives (bend angle optimization, cross-sectional shape preservation). Third, grid independence and experimental validation studies would support engineering applications and verify the reliability of the reshaping algorithm in preserving or modifying specific geometric features. Finally, the significant cross-section deformation observed in 3D pipes at high Reynolds numbers suggests additional geometric constraints may be needed to preserve desired cross-sectional profiles for manufacturing considerations.

While this study focuses on numerical validation through literature comparison, experimental validation would be valuable for engineering applications.

### Data and software availability

#### Underlying data

Zenodo: Data for SEROS publication. https://doi.org/10.5281/zenodo.7788202

This project contains the following underlying data:

• rhobarInletAvg_analysis (Summary of achieved Pressure Drop Reduction (PDR) of all cases.)
• pre-processing-cases.zip (Directories containing the following files directly generated with SEROS.)
– bestSeros (Iteration at PDR was highest.)
– config2D_L.xml (Case configuration for creation of data.)
– measurementsSeros0 (Fluid measurements taken at each reshaping iteration.)
– plbLog.dat (Log file generated during simulation execution.)
– rho*.ppm (Visualisation of fluid density fields at given iterations.)
– vtk*.vti (Fluid field at highest PDR available for some cases.)
• 2D_L_Re*_PDR.txt (Identical with measurementsSeros0 for each 2D case.)
• 3D_L_Re*_PDR.txt (Identical with measurementsSeros0 for each 3D case.)

Data are available under the terms of the Creative Commons Attribution 4.0 International license (CC-BY 4.0)

### Software availability

David Blacher. (2023). AIT-LKR/SEROS: First Release with full source code (v1.0-full-source-code). Zenodo. https://doi.org/10.5281/zenodo.7788368

The software is also available under a AGPL license here: https://github.com/AIT-LKR/SEROS
