# Supplementary material for: Optimisation of pipes with constant diameter using the heuristic optimality criterion
Source: Open Res Eur. 2026 Apr 9;3:156. Originally published 2023 Sep 21. [Version 2] doi: 10.12688/openreseurope.15943.2 (PMC13146464; doi:10.12688/openreseurope.15943.2)
Supplement: Peer review history

# Reviewer report: Reviewer response for version 2

**DOI:** 10.21956/openreseurope.25194.r72105

**Published:** 2026-05-05

**Authors:** Desejo Filipeson Sozinando

**Affiliations:** 1 Vaal University of Technology, Vanderbijlpark, South Africa

Most of the key issues raised in the initial review have been appropriately addresed in the revised version, in particular the method’s performance at higher Reynolds numbers and the need for methodological clarity. The manuscript now presents improved technical clarity, alongside a more comprehensive discussion and well-defined future work. Minor issues remain but do not affect the overall scientific quality.

Is the study design appropriate and does the work have academic merit?

Yes

Is the work clearly and accurately presented and does it cite the current literature?

Partly

If applicable, is the statistical analysis and its interpretation appropriate?

Yes

Are all the source data underlying the results available to ensure full reproducibility?

Yes

Are the conclusions drawn adequately supported by the results?

Partly

Are sufficient details of methods and analysis provided to allow replication by others?

Partly

Reviewer Expertise:

Vibration Analysis, Fluid-Structure Interaction, Mathematical Modelling, FEA/CFD, Machine Learning.

I confirm that I have read this submission and believe that I have an appropriate level of expertise to confirm that it is of an acceptable scientific standard.

# Reviewer report: Reviewer response for version 2

**DOI:** 10.21956/openreseurope.25194.r72651

**Published:** 2026-04-22

**Authors:** Sarmad A Ali

**Affiliations:** 1 University of Babylon, Babylon, Iraq

1- Revise the title to clearly reflect the scientific novelty (constant diameter constraint) and highlight the key finding related to Re = 40.

 2- Improve the abstract by clearly stating the research gap and problem, including study limitations (especially at high Reynolds numbers), and avoiding overgeneralization to turbulent flows.

 3- Update the introduction with recent references (≥50% from the last 5 years) and better emphasize the originality and research gap.

 4- Strengthen the introduction by adding real industrial application examples to justify the importance of the study.

 5- Expand the description of the Lattice Boltzmann Method (LBM), including justification for its use over conventional CFD, with discussion of its advantages and limitations.

 6- Clearly state the model assumptions (Newtonian fluid, laminar flow, isothermal conditions) and discuss their impact on generalizability.

 7- Provide stronger justification for the selection of numerical parameters (velocity, viscosity, relaxation time, adaptation time).

 8- Conduct a grid independence study to ensure the reliability and robustness of the results.

 9- Perform a sensitivity analysis for key parameters (e.g., f _e ,_ f _s_) and evaluate their effect on convergence and stability.

 10- Improve the mathematical model presentation by adding deeper physical interpretation of equations and ensuring consistency of symbols.

 11- Clarify the optimization algorithm (erosion/sedimentation), justify parameter choices, and define convergence criteria more rigorously.

 12- Enhance the results section with detailed physical analysis (e.g., shear stress distribution, flow patterns, vortex structures).

 13- Provide a clear scientific explanation for the superior performance at Re = 40 and the reduced effectiveness at Re ≥ 100.

 14- Support results with quantitative comparisons to previous studies and/or alternative optimization methods.

 15- Expand the discussion to include analysis of non-convergence and sensitivity to initial geometry.

 16- Address cross-sectional deformation in 3D cases and discuss its implications for practical/industrial applications.

 17- Explicitly state the methodological limitations within the discussion section.

 18- Revise the conclusions to avoid unsupported generalizations and ensure all claims are directly supported by results.

 19- Strengthen the future work section with specific directions (e.g., experimental validation, turbulent flow modeling, industrial applications).

 20- Update and improve the references by including recent studies in CFD and LBM, and ensure consistency in formatting, along with improving the clarity and quality of figures and tables.

Is the study design appropriate and does the work have academic merit?

Yes

Is the work clearly and accurately presented and does it cite the current literature?

Partly

If applicable, is the statistical analysis and its interpretation appropriate?

Partly

Are all the source data underlying the results available to ensure full reproducibility?

Partly

Are the conclusions drawn adequately supported by the results?

Yes

Are sufficient details of methods and analysis provided to allow replication by others?

Yes

Reviewer Expertise:

NA

I confirm that I have read this submission and believe that I have an appropriate level of expertise to confirm that it is of an acceptable scientific standard, however I have significant reservations, as outlined above.

# Reviewer report: Reviewer response for version 1

**DOI:** 10.21956/openreseurope.17217.r37318

**Published:** 2024-08-14

**Authors:** S Mahmood Mousavi

**Affiliations:** 1 Texas State University, San Marco, Texas, USA; 2 Seoul National University (Ringgold ID: 26725), Gwanak-gu, Seoul, South Korea

1-The numerical simulations conducted using the Lattice Boltzmann method should be described in detail, including the range of Reynolds numbers tested and any limitations or assumptions made.

 2- The method showed less effectiveness at higher Reynolds numbers. This is a significant limitation as many real-world applications operate in these regimes.

 3-There seems to be a lack of comparative analysis with other methods. It would be beneficial to see how this method stacks up against traditional approaches under similar conditions.

Is the study design appropriate and does the work have academic merit?

Yes

Is the work clearly and accurately presented and does it cite the current literature?

Partly

If applicable, is the statistical analysis and its interpretation appropriate?

Not applicable

Are all the source data underlying the results available to ensure full reproducibility?

Yes

Are the conclusions drawn adequately supported by the results?

Yes

Are sufficient details of methods and analysis provided to allow replication by others?

Partly

Reviewer Expertise:

Aerodynamic and reacting flow

I confirm that I have read this submission and believe that I have an appropriate level of expertise to confirm that it is of an acceptable scientific standard, however I have significant reservations, as outlined above.

# Reviewer report: Reviewer response for version 1

**DOI:** 10.21956/openreseurope.17217.r38577

**Published:** 2024-08-14

**Authors:** Desejo Filipeson Sozinando

**Affiliations:** 1 Vaal University of Technology, Vanderbijlpark, South Africa

The study aimed to minimize internal pressure drop in pipes and enhance the energy efficiency of fluid flow applications. The study proposed combining the heuristic optimization approach with a new geometrical constraint to assess pressure drop reduction solely by modifying the flow path while keeping the average diameter constant. This adds a novel aspect to the research, expanding on existing optimization techniques. The study conducts numerical simulations using the Lattice Boltzmann method with Reynolds numbers ranging from 40 to 500. However, the limited capability of the heuristic criterion to derive improved shapes with a minimum pressure drop is limited at Re ≥ 100 to achieving improvements which do not necessarily represent the optimal solution.

**General comments**

 The study appears to have academic merit, and the idea of the article is very interesting, but the authors have some shortcomings. The response and the suggested recommendation would define the acceptance of the manuscript.

**Specific Comments:**

 In order to have a better understanding of the manuscript, some aspects need to be clarified.

**1.         Highlighted of section 1:**

- The manuscript has about 17 references. Using 2023 as a baseline, the manuscript cited ten relevant works older than five years. In other words, 58% of the literature is older than five years. I recommend more than 50% of the current literature (not older than five years).

**2.         Highlighted of section 2.1:**

- How does the lattice Boltzmann method discretize the Navier-Stokes equations? What is the advantage in terms of accuracy and efficiency of the results over the traditional CFD method?

**3.         Highlighted of section 2.2:**

- Can you please justify the reason for the initialization of the velocity of the resultant new fluid nodes with zero after erosion? And how does this initialisation influence the fluid-solid interface's behaviour during reshaping?

**4.         Highlighted of section 3.1:**

- May the authors state one or two alternative ways of calculating wall-neighbouring nodes fractions ( *f _s_* and *f _e_*) affected by sedimentation and erosion, and what influence do they have on the outcomes of reshaping?
- In what ways does the strain rate, which guides the reshaping algorithm, differ between viscous and inviscid fluid domains? How does this difference impact the effectiveness of the algorithm?
- What role does viscosity play in determining the stability and convergence of the reshaping algorithm, especially in relation to the recommended fraction of affected nodes by Wang et al.? and how were these affected nodes determined?

**5.         Highlighted of section 3.3:**

- I would advise not including numbering in Figure 5, since the arrows already indicate the algorithm's articulation.

**6.         Highlighted of section 5:**

- In conclusion, it is stated that "the limited capability of the heuristic criterion to derive improved shapes with a minimum pressure drop is limited at Re ≥ 100 to achieving improvements which do not necessarily represent the optimal solution." I advise the author to recommend future research to address these limitations and improve the effectiveness of the reshaping algorithm for complex fluid flow scenarios. I also advise authors to recommend future work to validate or verify how reliable and accurate the reshaping algorithm is, especially in preserving desired geometric features.

Is the study design appropriate and does the work have academic merit?

Yes

Is the work clearly and accurately presented and does it cite the current literature?

Partly

If applicable, is the statistical analysis and its interpretation appropriate?

Yes

Are all the source data underlying the results available to ensure full reproducibility?

Yes

Are the conclusions drawn adequately supported by the results?

Partly

Are sufficient details of methods and analysis provided to allow replication by others?

Partly

Reviewer Expertise:

Vibration Analysis, Fluid Structure Interaction, Fracture Mechanics

I confirm that I have read this submission and believe that I have an appropriate level of expertise to confirm that it is of an acceptable scientific standard, however I have significant reservations, as outlined above.

# Reviewer report: Reviewer response for version 1

**DOI:** 10.21956/openreseurope.17217.r38568

**Published:** 2024-03-19

**Authors:** Hicham Ferroudji

**Affiliations:** 1 Boumerdes University, Boumerdes, Algeria

The manuscript addressed the flow through pipes with a constant diameter using the heuristic optimality criterion.

 It is an interesting manuscript, however, the following would improve the quality of the paper:

1. More details about the numerical model.
2. Comparison with experimental data from the literature to validate and test the accuracy of the model.
3. I would recommend to state clearly the originality of the work.

Is the study design appropriate and does the work have academic merit?

Yes

Is the work clearly and accurately presented and does it cite the current literature?

Yes

If applicable, is the statistical analysis and its interpretation appropriate?

I cannot comment. A qualified statistician is required.

Are all the source data underlying the results available to ensure full reproducibility?

Partly

Are the conclusions drawn adequately supported by the results?

Partly

Are sufficient details of methods and analysis provided to allow replication by others?

Partly

Reviewer Expertise:

Numerical modeling of internal fluid flows

I confirm that I have read this submission and believe that I have an appropriate level of expertise to confirm that it is of an acceptable scientific standard, however I have significant reservations, as outlined above.

# Reviewer report: Reviewer response for version 1

**DOI:** 10.21956/openreseurope.17217.r37322

**Published:** 2024-03-19

**Authors:** Somnath Roy

**Affiliations:** 1 Department of Mechanical Engineering, Indian Institute of Technology Kharagpur, Kharagpur, West Bengal, India

This article presents an interesting study for optimization of duct shapes. However, the major comment is regarding not presenting validation and grid independence study for the LBM method. The authors should add it in the revised manuscript.

 Also, can this method be extended on bend angle optimization as well as on higher Re flows?

Is the study design appropriate and does the work have academic merit?

Yes

Is the work clearly and accurately presented and does it cite the current literature?

Yes

If applicable, is the statistical analysis and its interpretation appropriate?

Yes

Are all the source data underlying the results available to ensure full reproducibility?

Partly

Are the conclusions drawn adequately supported by the results?

Yes

Are sufficient details of methods and analysis provided to allow replication by others?

Partly

Reviewer Expertise:

Computational Fluid Dynamics

I confirm that I have read this submission and believe that I have an appropriate level of expertise to confirm that it is of an acceptable scientific standard, however I have significant reservations, as outlined above.

# Reviewer report: Reviewer response for version 1

**DOI:** 10.21956/openreseurope.17217.r37314

**Published:** 2024-02-21

**Authors:** Sohail Rehman

**Affiliations:** 1 Islamia College Peshawar, Peshawar, Pakistan

*The main observations are listed below. The acceptance of the manuscript would depend on the revision. The author needs to provide a point by point response or provide a rebuttal.*

1. *How did you get the convergence of the numerical method LBM ?. *
2. *The authors have not explained, what type fluid is selected, what is motivation for selecting this fluid model, and what are the real world applications of this fluid under the conditions studied in this paper.*
3. *Provide mathematical expression for fluid volume. *
4. *As the study considers the constitutive modeling of a virtual fluid dynamics phenomena without validating the results from any physical experimental data. While modeling such virtual fluid dynamics phenomena, the authors take many assumptions to formulate the mathematical model, which may not be real and practical. Therefore, further explanation about physical configuration of problem should be included and the results should be discussed with relation to physical parameters involved in the model. The authors should also explain the physical situation is being modeled in the manuscript.*
5. *Explain the physical significance of boundary conditions and the reasoning behind their transformations.*
6. *Detailed comparison of the current results with results from other literature or commercial (CFD) codes are preferred to show the advantages and disadvantages of the presented model?*
7. *Please enrich the introduction section with related studies.*

Is the study design appropriate and does the work have academic merit?

Yes

Is the work clearly and accurately presented and does it cite the current literature?

Partly

If applicable, is the statistical analysis and its interpretation appropriate?

I cannot comment. A qualified statistician is required.

Are all the source data underlying the results available to ensure full reproducibility?

Yes

Are the conclusions drawn adequately supported by the results?

Partly

Are sufficient details of methods and analysis provided to allow replication by others?

Partly

Reviewer Expertise:

Fluid dynamics, Channel flow, Mathematical modeling

I confirm that I have read this submission and believe that I have an appropriate level of expertise to confirm that it is of an acceptable scientific standard, however I have significant reservations, as outlined above.

## References

[1] : Coupled energy and mass transport for non-Newtonian nanofluid flow through non-parallel vertical enclosure. *Ain Shams Engineering Journal* .2023;14(8) : 10.1016/j.asej.2022.102023 10.1016/j.asej.2022.102023

[2] : Modeling a non-Newtonian nanofluid flow between intersecting planes with slip mechanism. *Continuum Mechanics and Thermodynamics* .2023;35(1) : 10.1007/s00161-022-01162-z 61-80 10.1007/s00161-022-01162-z

[3] : Optimizing thermal characteristics and entropy degradation with the role of nanofluid flow configuration through an inclined channel. *Alexandria Engineering Journal* .2023;69: 10.1016/j.aej.2023.01.026 85-107 10.1016/j.aej.2023.01.026
